# ROS scavenging Mn_3_O_4_ nanozyme regulated immune microenvironment and affects intercellular interaction to promote wound healing in diabetes

**DOI:** 10.1093/rb/rbaf089

**Published:** 2025-08-23

**Authors:** Zhuoyuan Li, Ao Zheng, Chen Liang, Zhiyuan Mao, Tanjun Deng, Lingyan Cao, Chen Wang

**Affiliations:** Department of Plastic and Reconstructive Surgery, Shanghai Ninth People’s Hospital, Shanghai Jiao Tong University School of Medicine, Shanghai 200011, China; Department of Prosthodontics, Shanghai Ninth People’s Hospital, Shanghai Jiao Tong University School of Medicine, Shanghai 200011, China; Department of Plastic and Reconstructive Surgery, Shanghai Ninth People’s Hospital, Shanghai Jiao Tong University School of Medicine, Shanghai 200011, China; Department of Plastic and Reconstructive Surgery, Shanghai Ninth People’s Hospital, Shanghai Jiao Tong University School of Medicine, Shanghai 200011, China; Department of Neurosurgery, Shanghai Ninth People’s Hospital, Shanghai Jiao Tong University School of Medicine, Shanghai 200011, China; Department of Prosthodontics, Shanghai Ninth People’s Hospital, Shanghai Jiao Tong University School of Medicine, Shanghai 200011, China; Department of Plastic and Reconstructive Surgery, Shanghai Ninth People’s Hospital, Shanghai Jiao Tong University School of Medicine, Shanghai 200011, China

**Keywords:** diabetes, tri-manganese tetroxide nanozyme, wound healing, ROS scavenging, immune regulation

## Abstract

Diabetes-induced chronic wound healing poses significant clinical and economic challenges. In the pathological context of diabetic wounds, the accumulation of reactive oxygen species (ROS) and inflammatory factors is exacerbated, impeding the transition of macrophages from the M1 to M2 phenotype, thereby leading to prolonged wound healing. Therefore, this study has developed an ultra-small tri-manganese tetroxide nanozyme with dual superoxide dismutase/catalase enzymatic activities, which exhibits excellent ROS scavenging performance. Under oxidative stress conditions, this nanozyme can alleviate mitochondrial damage and promote the transition of macrophages from the M1 to M2 phenotype, thereby mitigating the inhibition of cellular function caused by the inflammatory state through intercellular interactions. Furthermore, the application of this nanozyme *in vivo* has also contributed to the treatment of skin defects in streptozotocin-induced diabetic mice by alleviating inflammation and scavenging ROS. The dual-enzymatic nanozyme designed and prepared in this study, which scavenges ROS, can regulate the local immune microenvironment and intercellular interactions, providing a new strategy for the clinical treatment of diabetic wound healing.

## Graphical abstract

**Figure:**
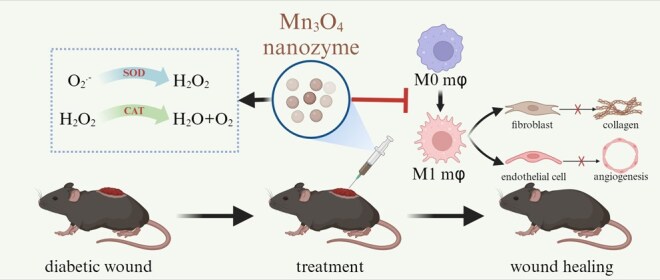

## Introduction

Diabetes is a global public health issue, with chronic hyperglycemia inducing pathological cascades that impair wound healing through persistent oxidative stress and dysregulated inflammation [1–4]. In diabetic wounds, elevated reactive oxygen species (ROS) levels disrupt extracellular matrix (ECM) remodeling through matrix metalloproteinase activation while amplifying pro-inflammatory cytokine production, collectively inhibiting the transition from inflammatory to proliferative healing phases [5–8]. Although endogenous antioxidant enzymes such as superoxide dismutase (SOD) and catalase (CAT) maintain ROS homeostasis under physiological conditions, their diminished activity in diabetic microenvironments exacerbates oxidative damage and perpetuates chronic inflammation [9–11]. Therefore, the introduction of nanomaterials with SOD/CAT enzyme-like activity is particularly reasonable for promoting diabetic wound healing.

Effective wound healing requires a substantial amount of extracellular components and the interaction of various cell types, such as immune cells, fibroblasts and endothelial cells [12, 13]. The polarization dynamics of macrophages critically regulate tissue repair processes [14, 15]. While M1 pro-inflammatory macrophages dominate early healing phases, timely transition to M2 reparative phenotypes facilitates angiogenesis and matrix deposition [16, 17]. However, diabetic hyperglycemia induces persistent M1 polarization through ROS-mediated NF-κB activation, triggering continuous inflammation at the wound site, ultimately leading to non-healing wounds or scarring [18]. Thus, therapeutic strategies promoting M2 polarization through ROS scavenging, therefore, represent a promising approach to reverse pathological inflammation.

Beyond macrophage reprogramming, effective wound healing requires coordinated interactions between immune cells, fibroblasts and endothelial cells. M2 macrophages secrete transforming growth factor-β1 (TGF-β1) and interleukin-10 (IL-10) that stimulate fibroblast-mediated collagen synthesis, while platelet-derived growth factor-β enhances endothelial angiogenesis [19, 20]. Recent studies demonstrate that M2-derived exosomes containing vascular endothelial growth factor and IL-1β can rescue angiogenesis in hyperglycemic conditions, highlighting the therapeutic potential of modulating macrophage paracrine signaling [21, 22]. Nevertheless, sustained ROS elevation in diabetic wounds disrupts these cellular interactions by inducing fibroblast senescence and endothelial dysfunction [23].

Nanozymes are a class of nanomaterials with intrinsic enzyme-like activities that can effectively catalyze enzyme substrates under mild conditions and exhibit catalytic efficiencies and enzyme reaction kinetics similar to those of natural enzymes [24–26]. Compared with biological enzymes, nanozymes can maintain 85% of their catalytic activity even under strong acid/base conditions (pH ≈ 2–10) or over a wide temperature range (≈4–90°C), holding great promise for clinical applications [27]. Recent studies have found that carefully designed nanozymes can act as SOD or CAT to regulate the body’s ROS levels, providing appropriate clues for oxidative stress-related diseases [28]. Tri-manganese tetroxide (Mn_3_O_4_) is a manganese-based nanozyme with manganese as its active center, which has attracted widespread attention due to its highly enhanced SOD-mimicking enzyme performance [29]. The inherent biocompatibility of manganese—an essential trace element in human antioxidant systems—further positions Mn_3_O_4_ as an ideal candidate for diabetic wound applications [30, 31].

**Figure 1.**
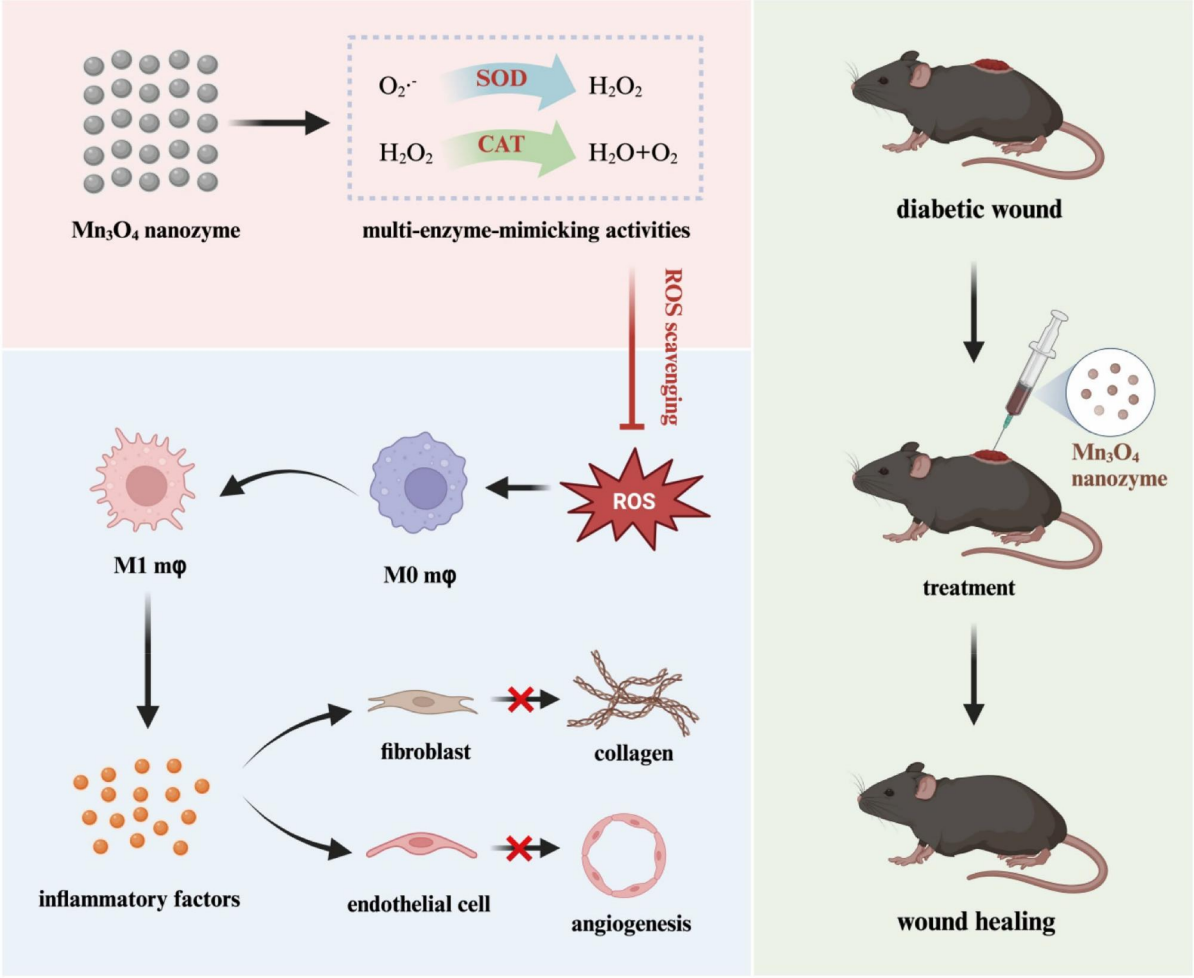
Schematic representation for the design strategy and applications of ROS scavenging Mn_3_O_4_ nanozyme regulated immune microenvironment and intercellular interaction to promote diabetes wound healing.

Therefore, this study aimed to prepare ultra-small nanostructured Mn_3_O_4_ nanozyme, explore their dual SOD/CAT enzyme activities, ROS scavenging ability and biocompatibility, investigate their regulatory effects on macrophage polarization, and examine their crosstalk effects on fibroblasts and vascular endothelial cells. Ultimately, the study will assess their application effectiveness in promoting wound healing in diabetic mice. By utilizing nanozyme, the study aimed to alleviate chronic inflammation in diabetic wounds, regulate the immune microenvironment and intercellular interactions and provide novel therapeutic strategies for promoting diabetic wound healing (Figure 1).

## Materials and methods

### Materials

Manganese acetate hydrate and methanol were purchased from Sinopharm Chemical Reagent Co., Ltd, China. The SOD enzyme activity assay kit, CAT enzyme activity assay kit, ABTS assay kit, mitochondrial membrane potential assay kit with JC-1 and DCFH-DA staining kit were purchased from Beyotime Biotechnology, China. IL-6 (Boster BA4339), CD86 (Abcam ab239075), CD206 (Abcam ab64693) and iNOS (Abcam ab136918) antibodies were used.

### Methods

#### Preparation of Mn_3_O_4_ nanozyme

A 1.225 g sample of manganese acetate was dissolved in 60 mL of methanol with magnetic stirring until fully dissolved. The mixture was then transferred to a 100 mL hydrothermal autoclave and heat treated at 120°C for 24 h. After cooling to room temperature, the product was washed with deionized water three times. The resulting dark brown precipitate was dried and ground into a powder.

#### Characterization of Mn_3_O_4_ nanozyme

##### Transmission electron microscope

The Mn_3_O_4_ nanozyme was dispersed in ethanol and sonicated for 5 min. An appropriate amount was then placed on a copper mesh and allowed to dry before being examined under a transmission electron microscope.

##### Scanning electron microscope

To observe the morphology and elemental composition of Mn_3_O_4_ nanozyme, scanning electron microscope images and energy dispersive spectroscopy (EDS) mappings were obtained using Zeiss Merlin Compact.

##### Particle size distribution and zeta potential

The particle size and zeta potential of Mn_3_O_4_ nanozyme were investigated using a particle size analyzer (Nicomp 380, PSS, USA).

##### X-ray diffraction

The Mn_3_O_4_ nanozyme was pressed into pellets and placed on glass slides. The sample was scanned at a rate of 2°/min using Cu-Kα X-ray with a tube voltage of 40 kV and a current of 40 mA. The crystalline structure was analyzed using an X-ray diffraction (XRD) instrument.

##### Fourier transform infrared spectroscopy

A suitable amount of the sample and dry potassium bromide powder were ground together in a mortar. The mixture was pressed into a transparent pellet, and the infrared spectra were collected.

##### X-ray photoelectron spectroscopy

To analyze the elemental composition and atomic valence states of the synthesized Mn_3_O_4_ nanozyme, powder samples were pressed into pellets, placed on the sample stage, vacuumed, and analyzed using an X-ray photoelectron spectroscopy (XPS) instrument. Full and fine spectra were collected for testing.

#### Enzyme activity of Mn_3_O_4_ nanozyme

A concentration gradient (0–128 μg/mL) of Mn_3_O_4_ nanozyme solution was prepared using the serial dilution method for enzyme activity assays.

##### SOD activity assay

SOD activity was determined by colorimetric analysis of the WST-8 product. The working solution was mixed with different concentrations of the Mn_3_O_4_ nanozyme solution, incubated at 37°C for 30 min, and the absorbance was measured at 450 nm using a microplate reader.

##### CAT activity assay

According to the manufacturer’s instructions, the sample solution, catalase assay buffer, and H_2_O_2_ solution were thoroughly mixed and reacted at 25°C for 5 min. After adding the color development reagent, the absorbance at 520 nm was measured after incubation at 25°C for 15 min.

##### Total antioxidant capacity (ABTS assay)

The working solution was mixed with different experimental groups, a blank control [phosphate buffered saline (PBS)] and a standard group (Trolox standard solution) and incubated at room temperature for 5 min. Absorbance at 734 nm was measured, and the total antioxidant capacity of the nanozyme was calculated from the standard curve.

#### Biocompatibility of Mn_3_O_4_ nanozyme

##### CCK-8 cell proliferation and toxicity assay

Mouse macrophage cells (RAW 264.7), mouse fibroblasts (L929), and human umbilical vein endothelial cells (HUVECs) were seeded in 96-well plates at 5 × 10^3^ cells per well. Different concentrations of Mn_3_O_4_ nanozyme solutions were added and incubated for 24 h. According to the product instructions, 100 µl of serum-free medium containing 10% CCK-8 solution was added to each well. After incubating for 1 h, absorbance at 450 nm was measured using a microplate reader.

##### Live-dead staining

RAW 264.7, L929, and HUVECs were seeded in confocal dishes at 5 × 10^4^ cells per well. After cell attachment, different concentration gradients of Mn_3_O_4_ nanozyme were added to stimulate the cells for 12 h. One milliliter of Calcein AM/PI staining solution was added, and the cells were incubated at 37°C in the dark for 30 min. The staining was observed using a confocal laser scanning microscope (CLSM).

##### The hemocompatibility of Mn_3_O_4_ nanozyme

Briefly, anticoagulated whole blood was acquired from mice. Anticoagulated whole blood was centrifuged (1000 rpm, 10 min), and erythrocytes from the pellet were diluted to 5% (v/v) in PBS. In a 48-well plate, 500 μL of nanozyme samples were mixed with 500 μL erythrocyte solution and incubated at 37°C for 1 h under agitation. Controls included 0.1% Triton X-100 (positive) and PBS (negative). Post-incubation, samples were centrifuged (1000 rpm, 10 min), and hemolysis was visually documented. Supernatant absorbance at 540 nm was measured using a microplate reader (Safire, TECAN, Switzerland). The hemolysis ratio was calculated with the following formula: Hemolysis ratio (%) = (A_S_ − A_NC_)/(A_PC_ − A_NC_) × 100%. Where the A_S_ represented the absorbance value of the sample supernatant, A_PC_ represented the absorbance value of the Triton X-100 positive control and A_NC_ represented the absorbance value of PBS.

#### Photothermal performance and *in vitro* antibacterial capability of Mn_3_O_4_ nanozyme

The photothermal performance of Mn_3_O_4_ nanozyme was evaluated by an 808 nm near-infrared (NIR) laser. Mn_3_O_4_ nanozyme (10 μg/mL) was investigated by irradiating with an 808 nm NIR laser (2.0 W cm^−2^, 300 s), with PBS as a control. Temperature changes were monitored by an infrared thermal imager (Fotric 280, China).

The antibacterial activity of Mn_3_O_4_ nanozyme against *Staphylococcus aureus* (*S. aureus*, ATCC 29213) and *Escherichia coli* (*E. coli*, ATCC 25922) was investigated *in vitro* using the spread plate method. A 200-μL bacteria suspension (1 × 10^4^ CFU/mL) was co-cultured with Mn_3_O_4_ nanozyme solution (10 μg/mL) for 6 h at 37°C with or without NIR irradiation (808 nm, 2.0 W cm^−2^, 10 min). A 100-μL bacterial solution was diluted and spread onto LB agar plates. After incubation at 37°C overnight, the bacterial colonies on the plates were photographed and the number of CFUs was counted using ImageJ to evaluate the inhibitory effect of bacteria. The bacterial suspension treated by PBS with or without NIR irradiation served as the control group.

#### Effect of Mn_3_O_4_ nanozyme on intracellular ROS scavenging in macrophages

RAW 264.7 cells (5 × 10^4^ cells) were seeded in confocal dishes. After cell attachment, experimental conditions were applied for 12 h. Intervention without the addition of Mn_3_O_4_ nanozyme and H_2_O_2_ was the control group (CTR group), the addition of 10 μg/mL of Mn_3_O_4_ nanozyme was the Mn_3_O_4_ group, and for the macrophages under normal culture, only 400 μM of H_2_O_2_ was added for the H_2_O_2_ group and 10 μg/mL of Mn_3_O_4_ nanozyme was added while adding the same concentration of H_2_O_2_ as the above, for the Mn_3_O_4_+H_2_O_2_ group. DCFH-DA staining reagent was added, and the cells were incubated at 37°C for 30 min. The results were observed and recorded using CLSM.

#### Effect of Mn_3_O_4_ nanozyme on mitochondrial oxidative stress in macrophages

##### Mitochondrial membrane potential assay

RAW 264.7 cells (5 × 10^4^ cells) were seeded in confocal dishes. Experimental grouping was performed as discussed in the section “Effect of Mn_3_O_4_ nanozyme on intracellular ROS scavenging in macrophages.” The cells were stained using the JC-1 assay kit, and the results were observed using a confocal fluorescence microscope.

##### Mitochondrial TEM

RAW 264.7 cells (5 × 10^4^ cells) were seeded in 6-well plates. Experimental grouping was performed as discussed in the section “Effect of Mn_3_O_4_ nanozyme on intracellular ROS scavenging in macrophages.” Cells were fixed with 2% glutaraldehyde at 4°C for 8 h and then with 1% osmium tetroxide for 1 h. After dehydration, the cells were observed under a TEM, and images were recorded.

#### Effect of Mn_3_O_4_ nanozyme on macrophage polarization

##### Real-time quantitative polymerase chain reaction

RAW 264.7 cells (5 × 10^4^ cells) were seeded in 6-well plates. After attachment, experimental conditions were applied for 12 h. Intervention without the addition of Mn_3_O_4_ nanozyme and H_2_O_2_ was the CTR group, and for the macrophages under normal culture, only 400 μM of H_2_O_2_ was added for the H_2_O_2_ group and 10 μg/mL of Mn_3_O_4_ nanozyme was added while adding the same concentration of H_2_O_2_ as the above, for the Mn_3_O_4_+H_2_O_2_ group. RNA was extracted using Trizol and analyzed for the expression of relevant genes using real-time quantitative polymerase chain reaction (RT-qPCR) (primer sequences in Supplementary Table S1).

##### Immunofluorescence staining

RAW 264.7 cells (5 × 10^4^ cells) were seeded in confocal dishes. After attachment, experimental conditions were applied for 12 h. Experimental grouping was done as above; cells were stained with CD206 (Abcam ab64693), iNOS (Abcam ab136918) and nuclei were stained with 4′,6-diamidino-2-phenylindole (DAPI). The results were observed using CLSM.

##### Western blot (WB) assays

RAW 264.7 cells (5 × 10^4^ cells) were seeded in confocal dishes. After attachment, experimental conditions were applied for 12 h. Experimental grouping was done as above; cells were lysed with Radio Immunoprecipitation Assay  Lysis buffer (Beyotime, China) containing protease inhibitors, and the supernatants were obtained by centrifugation. The protein concentration was determined by a Bicinchoninic Acid (BCA) Protein Assay Kit. Then the supernatants were separated by SDS-PAGE gel electrophoresis and transferred to polyvinylidene fluoride membranes (Millipore, Billerica, MA, USA). Next, the membranes were blocked by 5% BSA at room temperature and incubated with the primary antibodies at 4°C overnight. The primary antibodies used in this study include antibodies against CD206 (Abcam ab64693), iNOS (Abcam ab136918) and GAPDH (Proteintech, USA). After incubation with the corresponding secondary antibodies for 1 h at room temperature, the membranes were washed with Tris-buffered saline-Tween (TBS-T, Solarbio, China), and the target proteins were detected using an e-Blot Touch Imager.

##### Transcriptomic sequencing analysis

RAW 264.7 cells (5 × 10^6^ cells) were seeded in 10-cm culture dishes. After attachment, experimental conditions were applied for 12 h. RNA was extracted using Trizol, cDNA libraries were constructed and sequencing was performed on the Illumina HiSeq 4000 platform. RNA sequencing data and differentially expressed genes were analyzed using Ballgown software and annotation databases.

#### Effect of Mn_3_O_4_ nanozyme on cellular crosstalk

RAW 264.7 cells (5 × 10^4^ cells) were seeded in confocal dishes. After cell attachment, experimental conditions were applied for 12 h. Experimental grouping was performed as discussed in the section “Effect of Mn_3_O_4_ nanozyme on intracellular ROS scavenging in macrophages.” The supernatants from each group were collected, centrifuged at 1000 rpm for 5 min and an equal volume of fresh complete medium was added as conditioned media (CM). This was then added to L929 cells and HUVECs for co-culture. CCK-8 and Live-Dead assays were then performed as described above.

##### Effect on fibroblast cell migration

L929 cells (5 × 10^4^ cells) were seeded in 6-well plates. After attachment, CM from different groups were added for 12 h. Scratches were made using a 200 µL pipette tip, and dulbecco's modified eagle medium (DMEM) containing 2% serum was added. Migration was observed at 0, 6, 12 and 24 h, and images were captured.

##### Effect on endothelial cell migration and tube formation

HUVECs (5 × 10^4^ cells) were seeded in 6-well plates. After attachment, CM from different groups were added for 12 h. DMEM containing 2% serum was added, and tube formation was observed at 0, 6, 12 and 24 h. Images were captured.

HUVECs (5 × 10^4^ cells) were seeded in 6-well plates. After attachment, CM from different groups were added for 12 h. After centrifugation and resuspension, cells were added to 96-well plates containing Matrigel at a density of 3 × 10^4^ cells per well. After incubating at 37°C for 2 h, tube formation was observed and recorded. The results were analyzed by the ImageJ.

#### Therapeutic effect of Mn_3_O_4_ nanozyme on full-thick skin defects in diabetic mice

The animal experiments were approved by the Ninth People’s Hospital Affiliated to Shanghai Jiao Tong University School of Medicine’s Ethics Committee (Approval No: SH9H-2024-A987-1). Twenty-four 6-8-week-old male C57BL/6 mice were used in this study.

##### Diabetic mouse model

A diabetic model was established by intraperitoneal injection of 150 mg/kg streptozotocin (STZ) into C57BL/6 mice, and blood glucose levels were measured to confirm the model’s success with a fasting blood glucose level above 16.7 mmol/L.

The experimental animals were randomly allocated into four distinct groups as follows: (1) The non-diabetic CTR received daily intraperitoneal injections of PBS in normal mice; (2) the diabetic control group (DM) was established through PBS administration in streptozotocin-induced diabetic mice; (3) the nanozyme treatment group (Mn_3_O_4_) involved normal mice receiving 10 mg/mL Mn_3_O_4_ nanozyme suspension via daily intraperitoneal injection; (4) the therapeutic intervention group (DM+Mn_3_O_4_) consisted of diabetic mice administered with 10 mg/mL Mn_3_O_4_ nanozyme.

##### Skin defect model

Mice were anesthetized, and a full-thickness skin defect (6 mm in diameter) was created on the back after depilation. Either 10 mg/mL of Mn_3_O_4_ nanozyme or no treatment was injected locally into the defect area. Photos and images were taken on days 0, 4, 7, 10 and 14 after model construction.

##### *In vivo* biosafety

After 14 days, the major organs (heart, liver, spleen, lung and kidney) of mice were harvested and stained with H&E to evaluate the systemic toxicity of Mn_3_O_4_ nanozyme.

##### Histological analysis

Sections were collected on days 7 and 14, paraffin-embedded, and stained with hematoxylin-eosin (H&E) and Masson’s trichrome stain. Immunohistochemistry staining for IL-6 (Boster BA4339), CD86 (Abcam ab239075), and CD206 (Abcam ab64693) was performed. Observations were made using a light microscope, and images were captured for analysis. For immunofluorescence staining, skin sections (days 7) were blocked in goat serum for 30 min at room temperature and incubated at 4°C overnight with the corresponding primary antibodies: anti-CD31 (Servicebio Co., Ltd, China). Next, they were incubated with corresponding secondary antibodies (Servicebio Co., Ltd, China) for 1 h, and the cell nuclei were stained with DAPI (Servicebio Co., Ltd, China) for 10 min in the dark. Finally, the immunofluorescence image was captured using CLSM.

#### Statistical analysis

Data are expressed as mean ± standard deviation (SD). Statistical analysis was performed using GraphPad Prism v9.0 (USA), and comparisons between groups were made using one-way analysis of variance. Unless otherwise specified, *P*-values were compared with the CTR group.

## Results

### Preparation and characterization of Mn_3_O_4_ nanozyme

**Figure 2.**
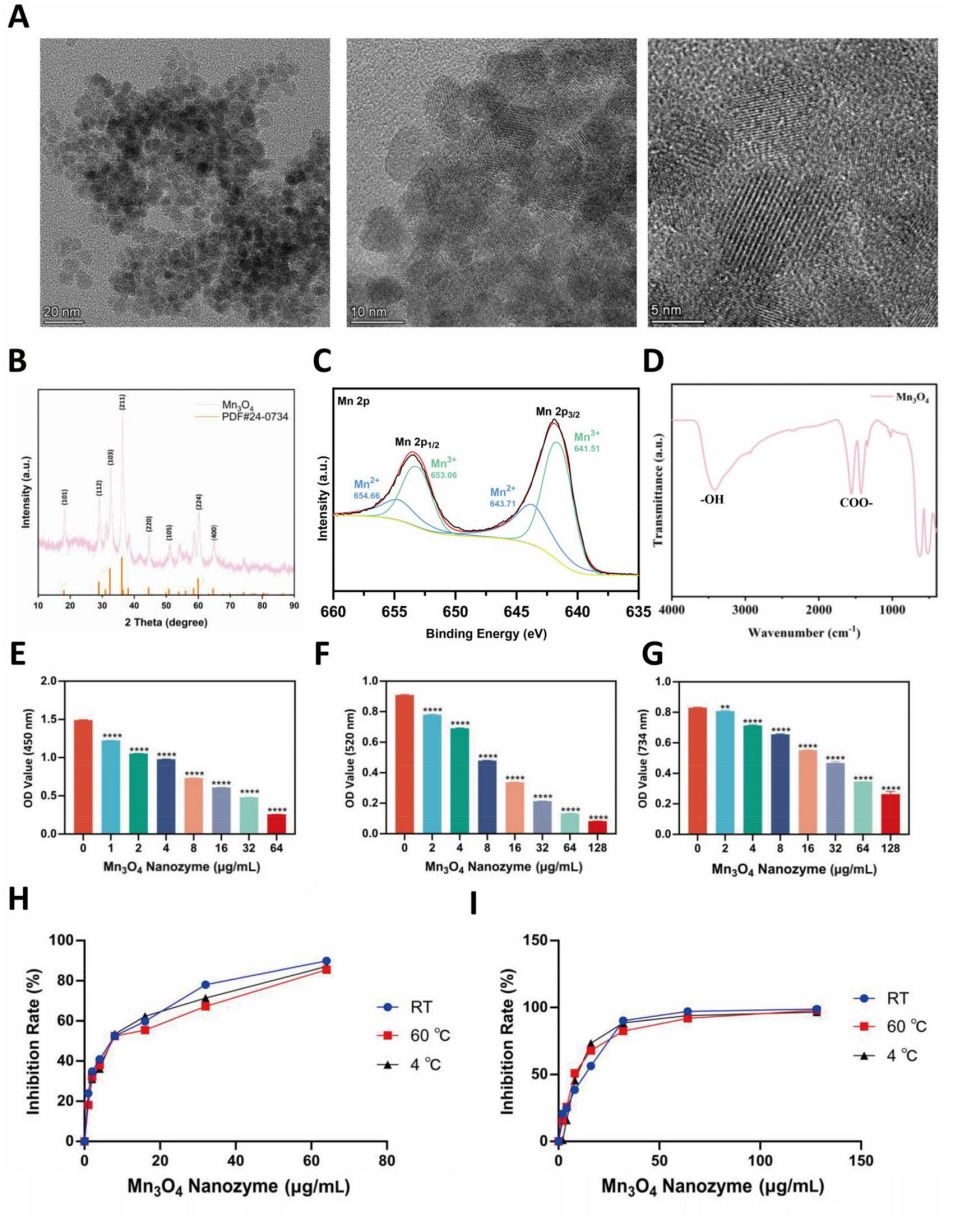
Characterization of synthesized Mn_3_O_4_ nanozyme. (**A**) HRTEM images, (**B**) XRD pattern, (**C**) XPS pattern and (**D**) FTIR pattern of the synthesized Mn_3_O_4_ nanozyme. Determination of enzyme activity of Mn_3_O_4_ nanozyme at different concentrations, (**E**) SOD activity detection, (**F**) CAT enzyme activity detection, (**G**) total antioxidant capacity detection, (**H** and **I**) enzyme activity of Mn_3_O_4_ nanozyme at different temperatures, (**H**) SOD activity detection, (**I**) CAT enzyme activity detection. Data are presented as mean ± SD. Statistical significance was determined using *t*-test (*n* = 3, ** *P* < 0.01, **** *P* < 0.0001).

In this study, brown-black Mn_3_O_4_ nanozyme were successfully prepared using a one-step hydrothermal method. TEM results, as shown in Figure 2A, demonstrated the successful preparation of ultra-small nanoparticles with a diameter of approximately 5 nm, exhibiting clear lattice fringes under high resolution. Particle size analysis (Supplementary Figure S1) revealed that the majority of Mn_3_O_4_ nanozyme exhibited diameters predominantly concentrated at 5 nm, consistent with observations from TEM results. EDS mapping analyses confirmed the presence of each element (Mn and O) in the nanozyme, providing direct evidence for the successful fabrication of the Mn_3_O_4_ nanozyme (Supplementary Figure S2). XRD analysis of the prepared nanozyme, shown in Figure 2B, reveals diffraction peaks that match the standard powder diffraction card for Mn_3_O_4_ (PDF#24-0734), confirming the pure phase of Mn_3_O_4_. XPS was used to characterize the valence states of the synthesized nanozyme (Figure 2C), with the Mn2p spectrum clearly showing peaks for Mn^2+^ and Mn^3+^. Fourier transform infrared spectroscopy (FTIR) results indicate the presence of some COO- and -OH groups, likely residues from acetic acid and methanol used in the preparation process (Figure 2D). The zeta potentials of Mn_3_O_4_ nanozyme in ddH_2_O and PBS were −9.87 and −9.11 mV, respectively, as shown in Supplementary Figure S3, which showed that the Mn_3_O_4_ nanozyme can form a stable dispersion.

Nanozyme, based on particles in the 1–100 nm range exhibiting new properties different from their macroscopic counterparts, is a class of mimetic enzymes that possess both the unique properties of nanomaterials and catalytic functions. The small and uniform size of the Mn_3_O_4_ nanozyme prepared in this project provides a good size foundation for exhibiting enzymatic activity.

### Enzymatic activity of Mn_3_O_4_ nanozyme

In this study, the SOD and CAT enzymatic activities of ultra-small Mn_3_O_4_ nanozyme were explored. As shown in Figure 2E, with increasing concentrations of Mn_3_O_4_ nanozyme, OD_450_ gradually decreases, indicating a reduction in the content of superoxide anions ($O_{2}^{-}$), which demonstrates that Mn_3_O_4_ nanozyme can effectively scavenge $O_{2}^{-}$, exhibiting SOD-like activity. Further testing using a CAT enzyme kit (Figure 2F) shows that as the concentration of Mn_3_O_4_ nanozyme increases, OD_520_ also decreases, representing a reduction in H_2_O_2_ concentration, indicating that the nanozyme exhibits CAT-like activity, effectively decomposing H_2_O_2_ into oxygen and water. Lastly, the total antioxidant capacity of Mn_3_O_4_ nanozyme was tested, with results similar to the aforementioned, showing a decrease in OD_734_ with the addition of nanozyme, further confirming the antioxidant capacity of the ultra-small Mn_3_O_4_ nanozyme (Figure 2G).

The usability of Mn_3_O_4_ nanozyme requires adaptability to different scenarios, making temperature stability particularly important. As shown in Figure 2H and I, the results indicate that samples incubated at different temperatures all exhibit good SOD and CAT enzymatic activities, with no statistical difference compared to the room temperature group, and the enzymatic activity of Mn_3_O_4_ nanozyme increases with concentration, indicating the good stability of Mn_3_O_4_ nanozyme, which can exhibit good enzymatic activity under different temperature conditions.

The oxidoreductase family, particularly the oxidase enzymes and antioxidative enzymes, such as SOD, CAT, and glucose oxidase, are employed in the wound healing process to successfully speed wound closure and skin regeneration [32]. The distinctive difference between diabetic ulcers and other common wounds is that the diabetic wound microenvironment possesses higher blood glucose levels. SOD and CAT are important antioxidant enzymes in the human body, playing a role in resisting oxidative stress within cells. Since diabetic wounds have high concentrations of free radicals and insufficient SOD expression, designing SOD-loaded biomaterials can effectively improve the hypoxic microenvironment of wounds and accelerate wound healing [33]. Meanwhile, CAT can decompose H_2_O_2_ into O_2_, thereby relieving oxidative stress and replenishing O_2_. Therefore, combining the two is beneficial to the faster healing of diabetic wounds [34]. The ultra-small Mn_3_O_4_ nanozyme prepared in this study exhibits combined SOD and CAT enzymatic activities, not only enhancing the antioxidant capacity of the material but also consuming harmful substances such as H_2_O_2_, thereby further reducing cellular oxidative stress and maintaining the stability of the intracellular environment to protect cells from damage caused by oxidative stress.

### Biocompatibility of Mn_3_O_4_ nanozyme

**Figure 3.**
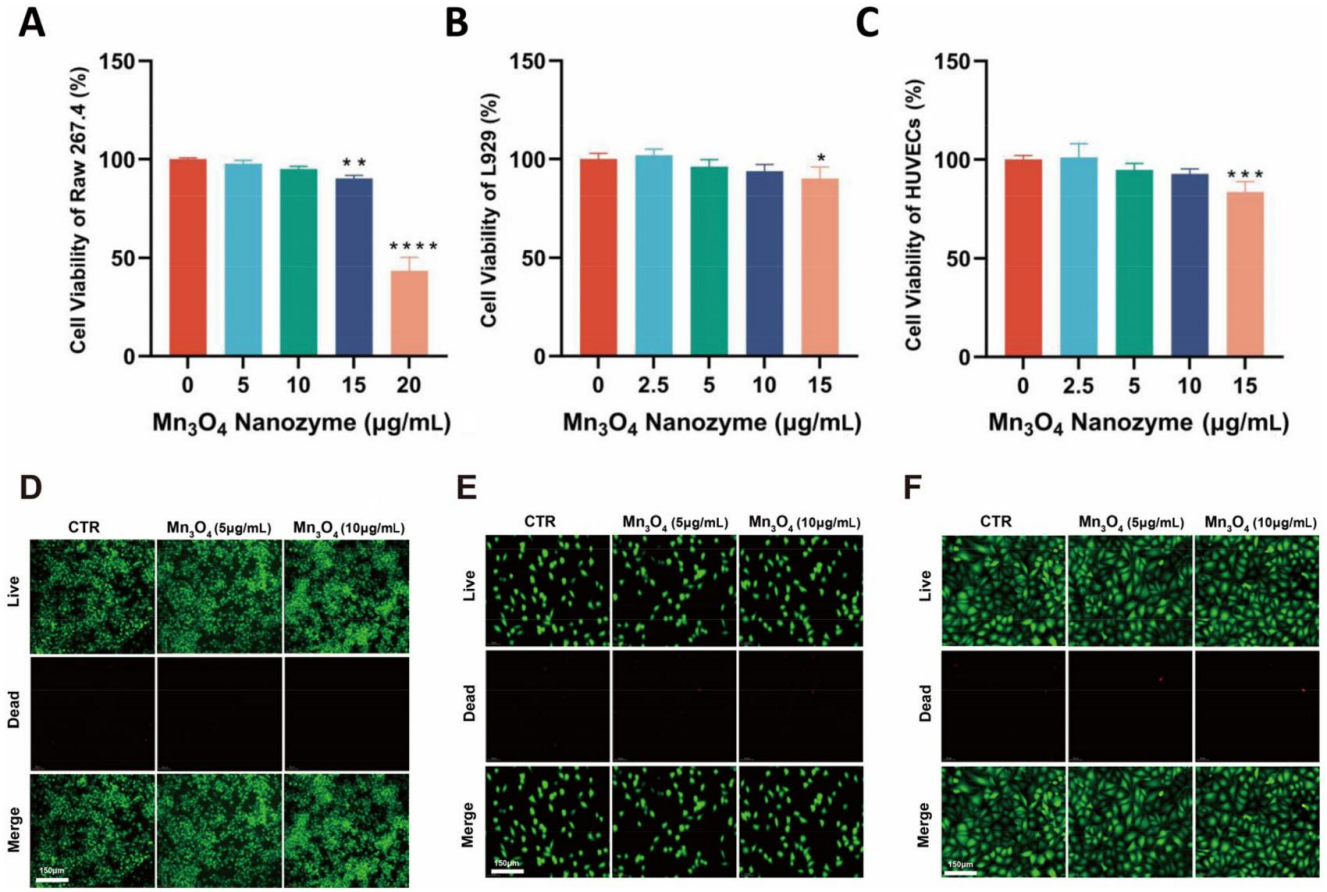
Cell viability in (**A**) RAW 264.7, (**B**) L929, (**C**) HUVECs after exposure of Mn_3_O_4_ nanozyme at different concentrations. Live-Dead assay in (**D**) RAW 264.7, (**E**) L929, (**F**) HUVECs after exposure of Mn_3_O_4_ nanozyme at different concentrations. Data are presented as mean ± SD. Statistical significance was determined using t-test (*n* = 3, *P < 0.05, **P < 0.01, ***P < 0.001, ****P < 0.0001).

The potential impact of nanomaterials on organisms, the environment and human health is gradually becoming a concern. Macrophages, fibroblasts and vascular endothelial cells are common cells in the process of skin wound healing. Cell viability was assessed using CCK-8 for the three cell types, with results shown in Figure 3A–C indicating that Mn_3_O_4_ nanozyme do not affect cell viability when the concentration is below 10 μg/mL, while cell viability begins to decline at concentrations of 15 μg/mL and above, and significantly inhibits cell activity at 20 μg/mL (*P* < 0.0001). Therefore, Mn_3_O_4_ nanozyme exhibits good biocompatibility when the concentration is below 10 μg/mL. For the three different cell types, after adding 5 μg/mL of Mn_3_O_4_ nanozyme, no significant cell death was observed, and even when the concentration was increased to 10 μg/mL, the red fluorescent signal representing cell death remained insignificant (Figure 3D–F). In summary, Mn_3_O_4_ nanozyme exhibited good biocompatibility at concentrations below 10 μg/mL, and thus, subsequent *in vitro* experiments were conducted using 10 μg/mL of nanozyme. The hemocompatibility of Mn_3_O_4_ nanozyme was further evaluated through hemolysis assays, revealing minimal hemolytic activity with rates consistently below 5%. These results (Supplementary Figure S4) demonstrate high hemocompatibility of Mn_3_O_4_ nanozyme with blood components. Collectively, the experimental evidence underscores the favorable biocompatibility of Mn_3_O_4_ nanozyme in *in vitro* assessments.

### Photothermal performance and *in vitro* antibacterial capability of Mn_3_O_4_ nanozyme

The photodynamic bactericidal strategy activated by NIR irradiation presents a promising therapeutic paradigm for managing diabetic skin defects, which exhibit heightened susceptibility to microbial colonization [35]. Based on our biocompatibility assessments, 10 μg/mL was selected as the optimal concentration. Under NIR irradiation (2.0 W cm^−2^), the 10 μg/mL Mn_3_O_4_ nanozyme solution exhibited rapid temperature elevation, reaching 40°C within 10 min, while the PBS control maintained a stable temperature (Supplementary Figure S5A).

This photothermal capability prompted further investigation of Mn_3_O_4_ nanozyme antibacterial activity *in vitro*. Following NIR irradiation (2.0 W cm^−2^, 600 s), both *S. aureus* and *E. coli* exhibited near-complete eradication with minimal residual colonies (Supplementary Figure S5B). Quantitative analysis (Supplementary Figure S5C and D) also confirmed the significant eradication efficacy. These results demonstrated the significant potential of Mn_3_O_4_ nanozyme for bactericidal applications, particularly in infected diabetic wounds.

### ROS scavenging by Mn_3_O_4_ nanozyme *in vitro*

**Figure 4.**
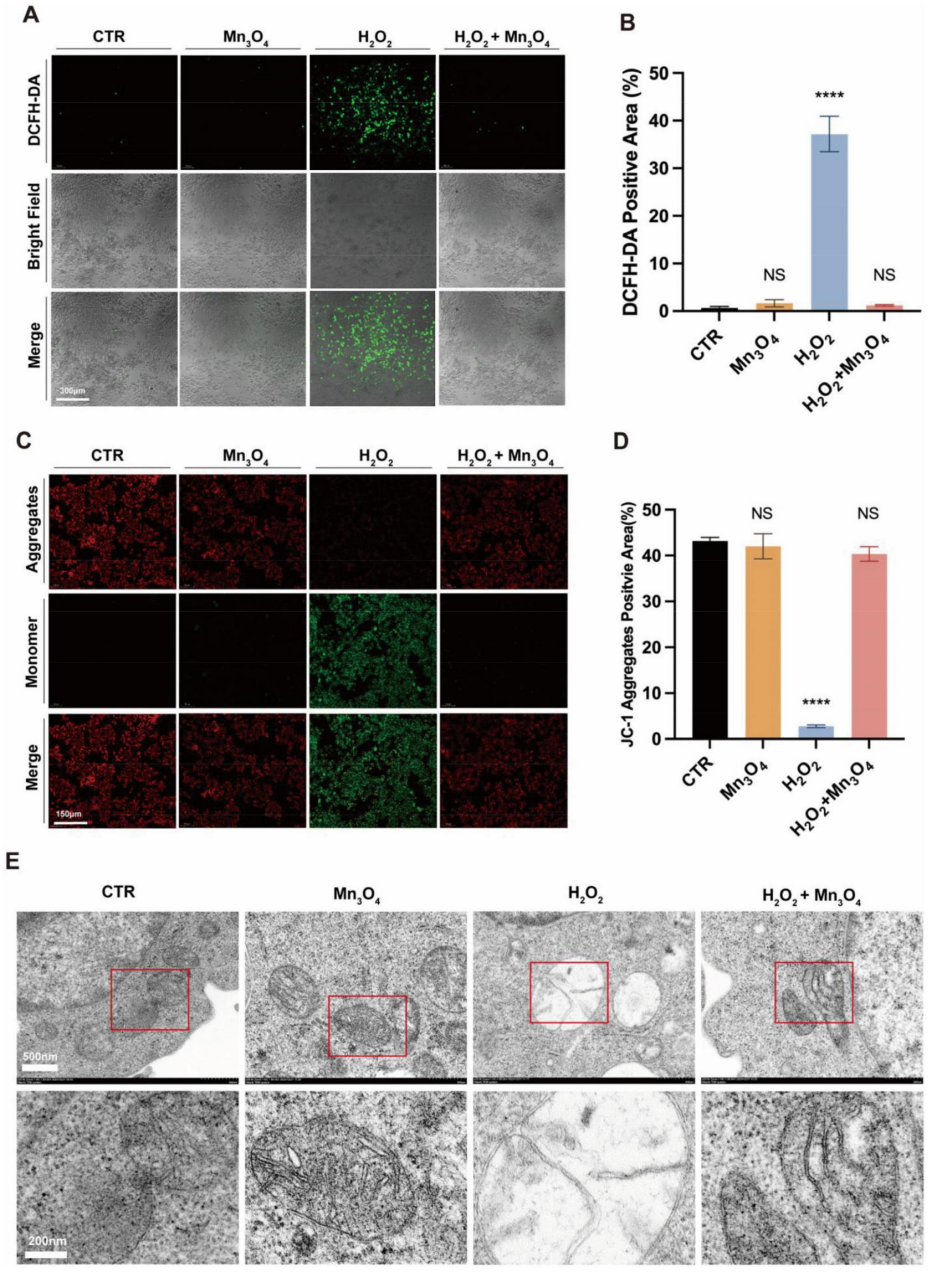
(**A**) Characterization and (**B**) quantitative analysis of clearance of intracellular reactive oxygen species by nanozyme. Protective effects on mitochondrial oxidative stress of nanozyme. (**C**) red fluorescence represents JC-1 polymer in mitochondria, and green fluorescence represents JC-1 monomer, (scale = 150 μm) and (**D**) quantitative analysis. (**E**) TEM observation of mitochondrial morphology. Data are presented as mean ± SD. Statistical significance was determined using *t*-test (*n* = 3, ns = no significance, ****P < 0.0001).

ROS scavenging in cells by nanozyme: cells treated with H_2_O_2_ showed increased ROS levels, while cells treated with Mn_3_O_4_ nanozyme exhibited fluorescent intensity of the ROS probe similar to the CTR group, maintaining low ROS levels, and quantitative fluorescence analysis showed no statistical difference between the two (*P* > 0.05), with a statistical difference compared to the H_2_O_2_ group (Figure 4A and B). For diabetic patients, excessive ROS in wounds can lead to oxidative stress, making it difficult for the wound to transition to the proliferative phase, and a prolonged inflammatory response can result in chronic non-healing wounds. The ultra-small Mn_3_O_4_ nanozyme prepared in this study also exhibited significant ROS scavenging ability within cells, alleviating oxidative stress damage.

Protective effect of nanozyme on mitochondrial oxidative stress: ultra-small Mn_3_O_4_ nanozyme that scavenges ROS can alleviate the oxidative stress state within cells, thereby protecting mitochondria. As shown in Figure 4C and D, the mitochondrial membrane potential is high in the CTR group and the Mn_3_O_4_ group; when 400 μM H_2_O_2_ is added, the mitochondrial membrane potential significantly decreases, while in the group treated with ultra-small Mn_3_O_4_ nanozyme, there is no significant difference in the expression of JC-1 polymers and monomers, and the mitochondrial membrane potential does not change significantly. Corresponding quantitative fluorescence analysis shows no statistical difference among the blank control group, material group and experimental group, indicating that the nanozymes themselves do not affect the mitochondrial membrane potential of macrophages, and the addition of nanozyme can effectively reverse intracellular oxidative stress and reduce mitochondrial damage when stimulated with H_2_O_2_.

Mitochondria are cellular organelles of varying sizes, shapes, such as spherical, rod-like or filamentous, and the intracellular oxidative stress state can easily damage mitochondria. Observation by biological transmission electron microscopy revealed that the number of mitochondrial cristae in the H_2_O_2_ group significantly decreased, with swelling observed, while the images in the H_2_O_2_+Mn_3_O_4_ group showed morphological features similar to the blank control group, indicating that Mn_3_O_4_ nanozyme can overcome the mitochondrial oxidative stress damage caused by H_2_O_2_ and effectively rescue mitochondria damaged by oxidative stress (Figure 4E). In summary, the results of these experiments demonstrate that the application of Mn_3_O_4_ nanozyme in the aforementioned *in vitro* cell experiments can effectively reduce cellular ROS levels and alleviate mitochondrial oxidative stress damage under oxidative stress conditions.

### Effect of Mn_3_O_4_ nanozyme on macrophage polarization

**Figure 5.**
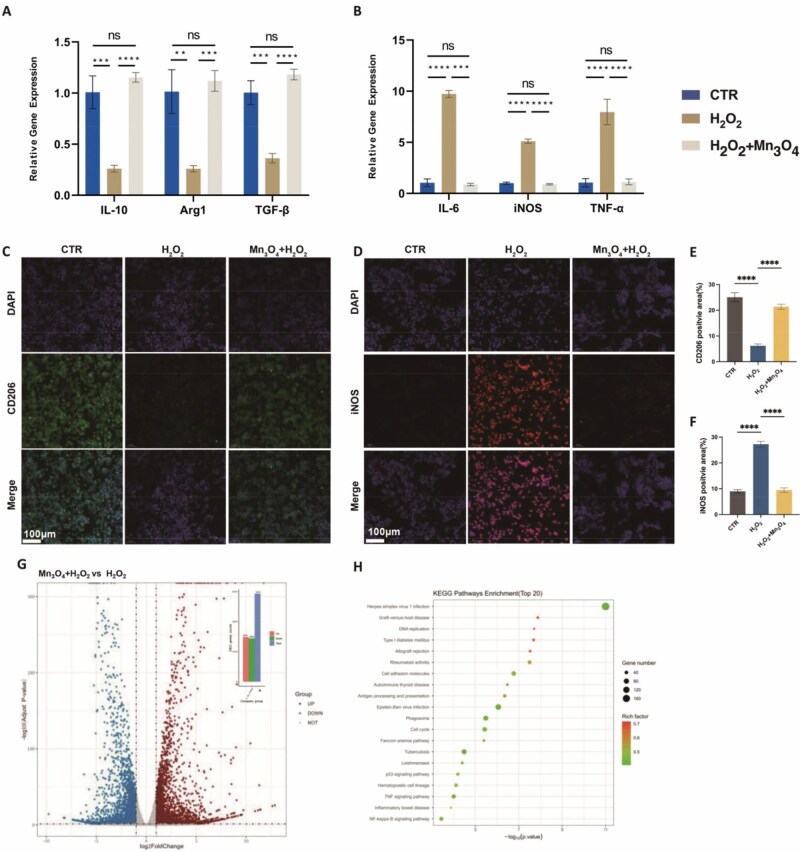
The polarization index of macrophages was detected by RT-qPCR: (**A**) M2 and (**B**) M1. Immunofluorescence of (**C**) CD206 and (**D**) iNOS, (**E** and **F**) quantitative analysis. (**G**) Different gene expression by bar graph and volcano plot, red represents upregulated genes and blue represents downregulated genes. (**H**) KEGG enrichment analysis of different expressed genes. Data are presented as mean ± SD. Statistical significance was determined using *t*-test (*n* = 3, ns = no significance, **P < 0.01, ***P < 0.001, ****P < 0.0001).

Macrophage polarization is closely related to the inflammatory state. The expression of M2 macrophage-associated genes IL-10, Arg-I and TGF-β showed the opposite trend, indicating that the addition of Mn_3_O_4_ nanozyme can alleviate the M1 polarization of macrophages induced by H_2_O_2_ and restore normal M1/M2 macrophage polarization conversion (Figure 5A). In contrast, RT-qPCR results showed that the M1 macrophage-associated markers tumor necrosis factor α (TNF-α), IL-6 and iNOS were significantly higher in the H_2_O_2_ group compared to the CTR group. Meanwhile, under the action of Mn_3_O_4_ nanozyme, inflammatory expression was effectively alleviated, with these three M1 macrophage markers significantly downregulated compared to the H_2_O_2_ group (Figure 5A). Additionally, WB analysis of macrophage marker proteins revealed distinct polarization patterns. The M2 marker CD206 exhibited significantly reduced expression in the H_2_O_2_ group compared to the CTR group, while the H_2_O_2_+Mn_3_O_4_ group showed partial restoration of CD206 levels (Supplementary Figure S6A). Conversely, the M1 marker iNOS displayed elevated expression in the H_2_O_2_ group compared to the CTR group, with H_2_O_2_+Mn_3_O_4_ exhibiting intermediate expression levels (Supplementary Figure S6B). Quantitative analysis confirmed that although both H_2_O_2_ and H_2_O_2_+Mn_3_O_4_ groups maintained lower CD206 expression than the CTR group, the nanozyme-treated group demonstrated significantly higher expression than H_2_O_2_ alone (Supplementary Figure S6C). These results indicated that H_2_O_2_ promotes M1 polarization, while Mn_3_O_4_ nanozyme mitigated this effect. Quantitative data further confirmed significantly lower iNOS expression in the H_2_O_2_+Mn_3_O_4_ group compared to the H_2_O_2_ group (Supplementary Figure S6D), collectively demonstrating the nanozyme capacity to counteract H_2_O_2_-induced M1 polarization.

In this study, immunofluorescence staining was used to detect polarization markers on the surface of macrophages. Compared to the H_2_O_2_ group, the signal for CD206 significantly increased after the addition of Mn_3_O_4_ nanozyme, indicating that the nanozyme can effectively prevent the effect of H_2_O_2_ on CD206 expression, although it cannot fully restore it to its original level (Figure 5C and E). The fluorescent expression of iNOS (Figure 5D and F) showed that the iNOS fluorescent signal area in the H_2_O_2_ group was nearly three times that of the blank control group. However, the addition of nanozyme effectively reversed this trend, with the iNOS fluorescent signal decreasing to levels similar to the CTR group, and no statistical difference between the two. This indicated that the scavenging of ROS by Mn_3_O_4_ nanozyme can effectively reduce the M1 polarization trend of macrophages induced by H_2_O_2_.

Cells treated with or without Mn_3_O_4_ nanozyme were collected for transcriptome sequencing. According to the sequencing results, the gene expression in macrophages significantly changed after the introduction of Mn_3_O_4_ nanozyme. As shown in Figure 5G, a total of 5854 genes were altered, with 2970 upregulated and 2884 downregulated (*P* < 0.05, |log2(fold change)| > 1). After the addition of Mn_3_O_4_ nanozyme, genes related to M1 polarization, such as IL-6, IL-1α, IL-1β and TNF, were significantly downregulated, indicating a notable reduction in M1-polarized macrophages upon Mn_3_O_4_ nanozyme treatment. Additionally, MMP9 was also significantly downregulated. The matrix metalloproteinase family is involved in degrading various protein components in the ECM and plays a detrimental role in the process of chronic wound healing in diabetes. As shown in Table S2, the |log2(fold change)| of these six genes was >1, with *P*-values ranging from 5.7425 × 10^−21^ to nearly 0 (statistical test result of 0), demonstrating highly significant statistical significance. Meanwhile, as shown in Figure 5H, KEGG (Kyoto Encyclopedia of Genes and Genomes) enrichment indicated that the NF-κB signaling pathway was activated after the addition of Mn_3_O_4_ nanozyme, suggesting that the effect of Mn_3_O_4_ nanozyme on macrophages by scavenging ROS may be mediated through the NF-κB signaling pathway.

### Interactions between macrophages and other cells

Abnormal M1 macrophages and impaired M1/M2 transition are associated with angiogenesis and epithelialization in diabetic wound healing [36]. The impaired polarization is closely associated with insufficient angiogenesis, as the anti-inflammatory M2 phenotype robustly supports vascular endothelial growth factor, a pivotal angiogenic source [37]. Conversely, M2 macrophages facilitate epithelialization through the secretion of epidermal growth factor and keratinocyte growth factor, while M1 macrophages impede fibroblast migration *via* TNF-α overexpression [38]. Collectively, M1/M2 polarization dynamically interacts with cellular and acellular components in diabetic wound healing, thus warranting further investigation into macrophage-mediated intercellular crosstalk.

#### Interactions between macrophages and fibroblasts

**Figure 6.**
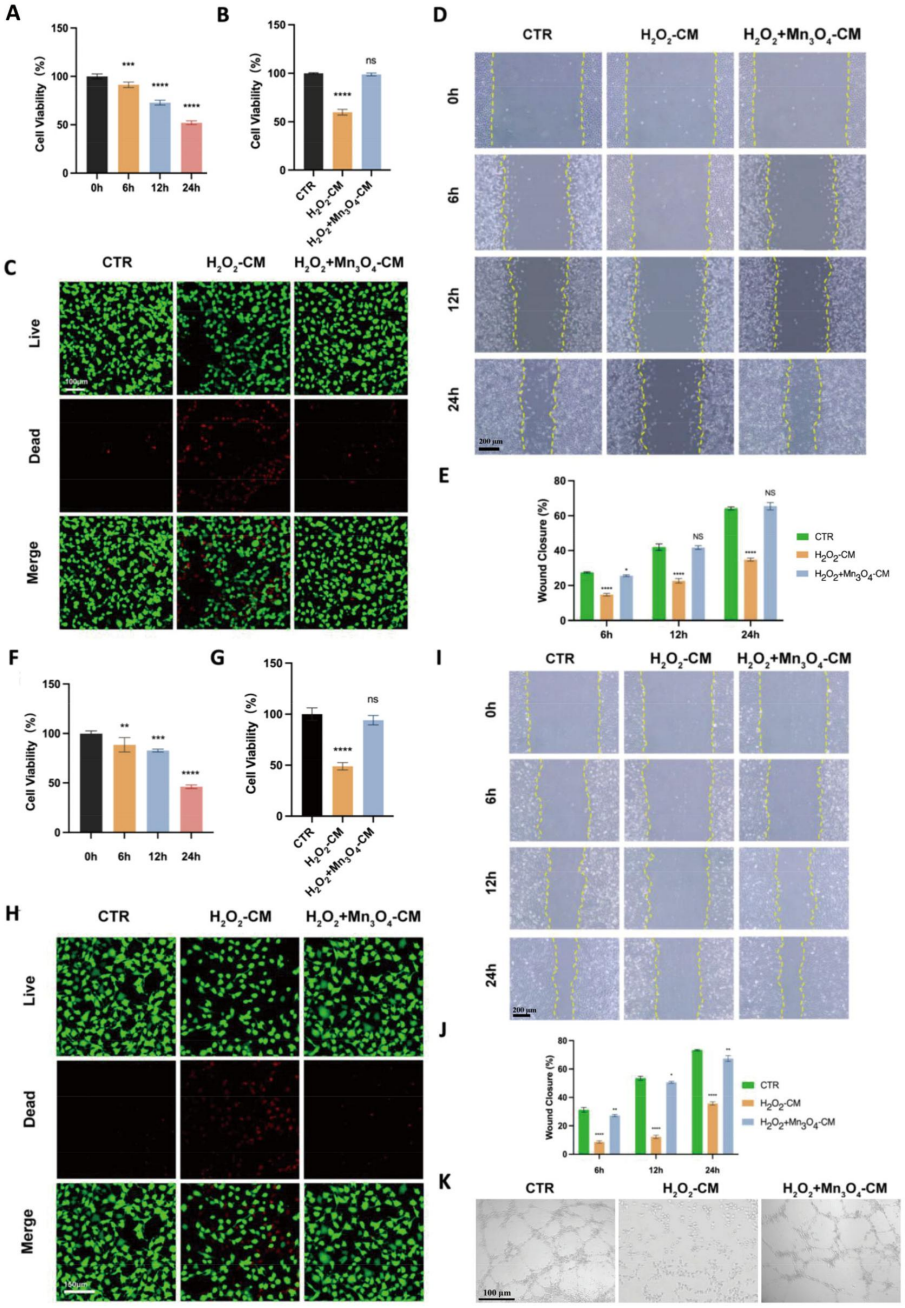
Intercellular interaction between macrophages and fibroblasts mediated by nanozyme. Effect of macrophage supernatant on fibroblasts under different culture mediums. (**A**) Fibroblast proliferation under H_2_O_2_-CM. (**B**) CCK test and (**C**) live-dead staining on fibroblast cultured by the supernatants from macrophages stimulated by different mediums. (**D**) Migration and (E) quantitative analysis. Effect of macrophage supernatant on HUVECs under different culture mediums. (**F**) HUVECs proliferation under H_2_O_2_-CM. (**G**) CCK test and (**H**) live-dead staining. (**I**) Migration and (**J**) quantitative analysis. Effect of macrophage supernatant on HUVECs under different conditions. Effects of macrophage supernatant on HUVECs (**K**) Tube formation assay. Data are presented as mean ± SD. Statistical significance was determined using t-test (*n* = 3, ns = no significance, **P < 0.01, ***P < 0.001, ****P < 0.0001).

The polarization of macrophages alters the local immune microenvironment, thereby affecting the biological behavior of other cells involved in wound healing. To determine whether supernatants from macrophage co-cultures affected fibroblast proliferation, we performed a CCK-8 assay on fibroblasts treated with these supernatants. It can be seen in Figure 6A that compared with the CM, the supernatant of the macrophage stimulated with H_2_O_2_ had a significant difference observed at 6 h after the addition of H_2_O_2_-CM, and the trend of proliferation inhibition was even more significant when the duration of supernatant addition reached 12 and 24 h, which indicated that the supernatant of macrophage stimulated with H_2_O_2_ had an inhibitory effect on fibroblasts proliferation. As shown in Figure 6B, compared with CM, the supernatant from macrophages stimulated by H_2_O_2_-CM inhibited the proliferation of fibroblasts, while H_2_O_2_+Mn_3_O_4_-CM did not show inhibition. Live-Dead staining results (Figure 6C) showed that compared with the CTR group, H_2_O_2_-CM had more dead cells, while the H_2_O_2_+Mn_3_O_4_ group had no significant dead cells.

As one of the key cells in the wound healing process, the migration ability of fibroblasts is also crucial. As shown in Figure 6D and E, under the influence of H_2_O_2_-CM, cell migration was impaired. At 24 h post-scratch, the healing rates of the CTR and H_2_O_2_+Mn_3_O_4_ groups had reached over 60%, while the H_2_O_2_ group was only about half of the former two (34.8%). This indicates that changes in macrophage polarization can promote the migration of L929 cells, thereby enhancing wound healing.

#### Interactions between macrophages and vascular endothelial cells

Vascular endothelial cells are also a fairly important cell type in the wound healing process. Figure 6F showed that compared with the CM, the H_2_O_2_-CM had an inhibitory effect on the proliferation of HUVECs, in which a significant difference was observed at 6 h after the addition of the H_2_O_2_-CM, and the tendency of the inhibition of the proliferation was more significant when the duration of the H_2_O_2_-CM addition reached 12 and 24 h, with the cell viability decreasing to 82.8% and 46.2 of that of the control group, respectively, which indicates that the hydrogen peroxide-stimulated macrophage supernatant had a significant effect on HUVECs proliferation. As shown in Figure 6G, compared with CM, H_2_O_2_+Mn_3_O_4_-CM did not inhibit the proliferation of HUVECs. Live-Dead staining results (Figure 6H) showed that compared with the CTR group, H_2_O_2_-CM had more dead cells, while the H_2_O_2_+Mn_3_O_4_ group had no significant dead cells, similar to the results with L929 cells. This indicates that the addition of Mn_3_O_4_ nanozyme can alleviate the inhibitory effect of H_2_O_2_ on cell proliferation.

This study also examined the migration ability of endothelial cells. The scratch assay results (Figure 6I and J) showed that at 24 h post-scratch, the healing rates of the CTR and H_2_O_2_+Mn_3_O_4_ groups had reached 73.3% and 67.4%, respectively, significantly higher than the 35.7% of the H_2_O_2_ group. This indicates that changes in macrophage polarization can promote the migration of HUVECs, thereby enhancing wound healing.

In addition to proliferation and migration, angiogenic capacity is also a common functional evaluation index for vascular endothelial cells. As shown in Figure 6K, under appropriate conditions, HUVECs in the CTR group formed tubular arrangements in the matrix gel, while the H_2_O_2_ group, influenced by the supernatant of macrophages treated with H_2_O_2_, showed significantly impaired tube formation. However, the H_2_O_2_+Mn_3_O_4_ group effectively formed tubes; quantitative analysis of junction number and total length revealed statistically significant differences between the H_2_O_2_+Mn_3_O_4_ and the H_2_O_2_ groups (Supplementary Figure S7), once again verifying that Mn_3_O_4_ nanozyme can effectively mitigate the adverse effects of H_2_O_2_-CM on endothelial cell function.

Combining the aforementioned results, it is clear that H_2_O_2_-CM has adverse effects on the proliferation and function of fibroblasts and endothelial cells, while the supernatant incubated with Mn_3_O_4_ nanozyme does not have such adverse effects. This indicates that Mn_3_O_4_ nanozyme can alter macrophage polarization, thereby affecting the biological behavior of other cells and eliminating the adverse effects of H_2_O_2_ on cells.

### Promotion of diabetic skin wound healing by Mn_3_O_4_ nanozyme

**Figure 7.**
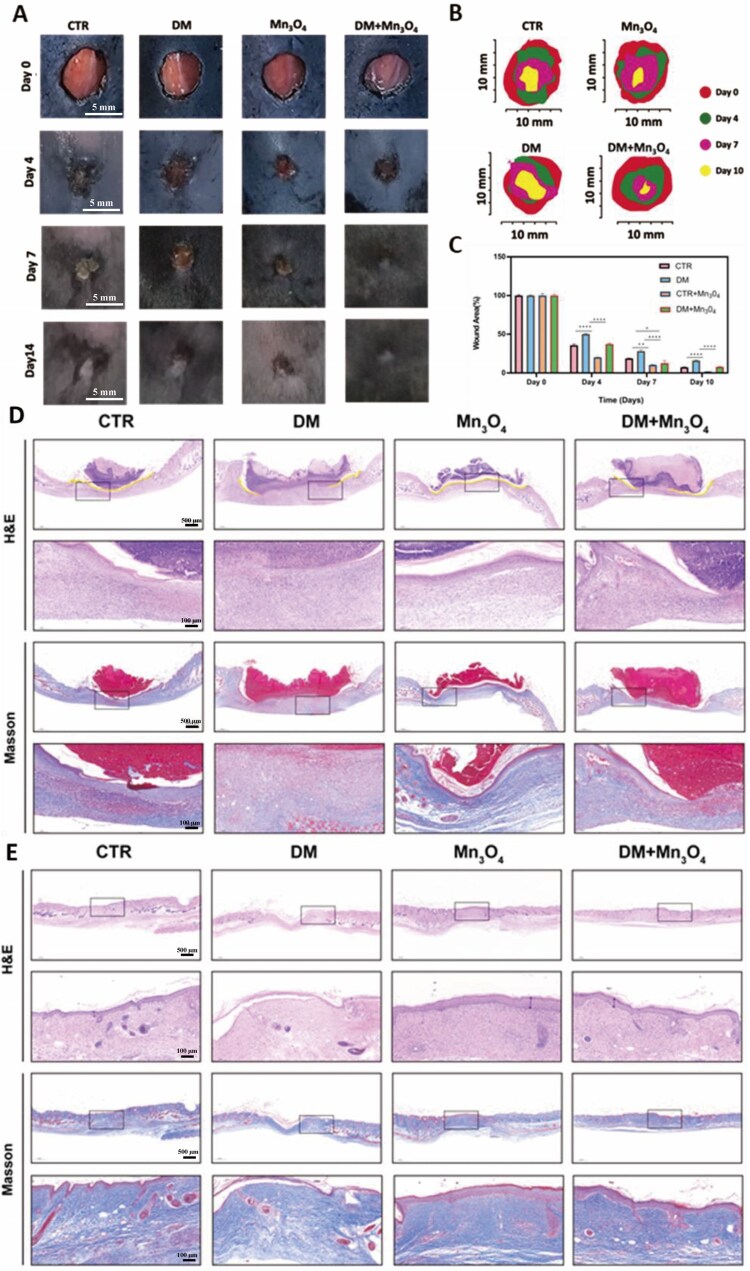
(**A**) Wound healing in CTR, DM, Mn_3_O_4_ and DM+Mn_3_O_4_ groups on day 0, day 4, day 7 and day 14. (**B**) Simulation diagram of wound healing in each group on different days. (**C**) Statistical results of wound healing relative area in each group on different days. (**D**) H&E and Masson staining in each group on day 7. The yellow lines indicate areas of epithelialization in H&E staining. (**E**) H&E and Masson staining in each group on day 14. Data are presented as mean ± SD. Statistical significance was determined using *t*-test (*n* = 3, *P < 0.05, ***P < 0.001, ****P < 0.0001).

Observations were made on days 0, 4, 7 and 10 after the creation of the full-thickness skin defect model. As shown in Figure 7A–C, over time, the Mn_3_O_4_ group showed faster healing, followed by the CTR group and the DM+Mn_3_O_4_ group, which showed similar overall trends. The DM group had the worst recovery. This indicates that Mn_3_O_4_ nanozyme alone promotes wound healing to some extent in non-diabetic conditions, which is closely related to its promotion of M2 polarization in macrophages, suggesting that it can promote wound healing by modulating the local immune microenvironment. Furthermore, the addition of Mn_3_O_4_ nanozyme in the diabetic model restored the healing of diabetic mice to levels comparable to normal mice, indicating that the nanozyme can also improve local oxidative stress damage in diabetes and promote normal wound healing *in vivo*.

The staining results on day 7 are shown in Figure 7D. H&E and Masson staining were performed, respectively, in which according to the results of H&E staining, it can be found that obvious scab tissue can be seen in each group under the microscope, and the structure of the granulation tissue consisting of neonatal capillaries, fibroblasts, and inflammatory cells is obvious, but there is a difference in inflammatory infiltration between the groups, and the inflammatory cells in the DM group are more numerous than the other three groups, which can be seen more obviously after turning up the magnification. The DM group had more inflammatory cells than the other three groups. On the other hand, the epithelialization of the trauma is also quite critical. The yellow line marks the part of the skin that was re-epithelialized after the skin defect, and it can be seen that the CTR group and the Mn_3_O_4_ group have formed complete and continuous epithelialization, while the remaining two do not yet have it, and there are obvious areas of skin lesions, and the sarcolemma under the squamous epithelial tissues is fractured and discontinuous; the tissues also have neutrophilic infiltration and large numbers of lymphocytes. Masson’s staining was mainly used to observe collagen deposition during wound healing, where blue color is the area representing positivity, and the results showed that at day 7, the Mn_3_O_4_ group had a higher amount of collagen, denser matrix deposition, and a higher number of collagen fibers, which was significantly higher than that of the remaining three groups, whereas the DM group had significantly fewer. The results of H&E and Masson staining on day 14 after modeling are shown in Figure 7E, where the degree of wound healing, level of collagen deposition, and even epithelialization increased in all groups. Unlike the degree of epithelialization at day 7, complete epithelialization was formed in the DM+Mn_3_O_4_ group, and the epidermis was seen to be gradually continuous and dense, the dermal tissue was complete, and complete squamous epithelial tissue was seen, and the thickness of the tissue was increased in all cases, and the fibroblasts transformed into fibroblasts with a large number of collagen fibers, whereas the DM group still did not have a complete formation, and these results illustrated that, at the histological level, the scavenging of ROS by Mn_3_O_4_ nanozyme ROS had a promoting effect on wound healing, and the results were basically the same as those of the macroscopic results. Finally, histopathological examination of major organs (heart, liver, spleen, lungs and kidneys) was performed *via* H&E staining on day 14 to validate the clinical safety of nanoparticles. Results demonstrated no observable pathological alterations across all experimental groups (Supplementary Figure S8), confirming excellent histocompatibility between the nanoparticles and vital organ tissues.

**Figure 8.**
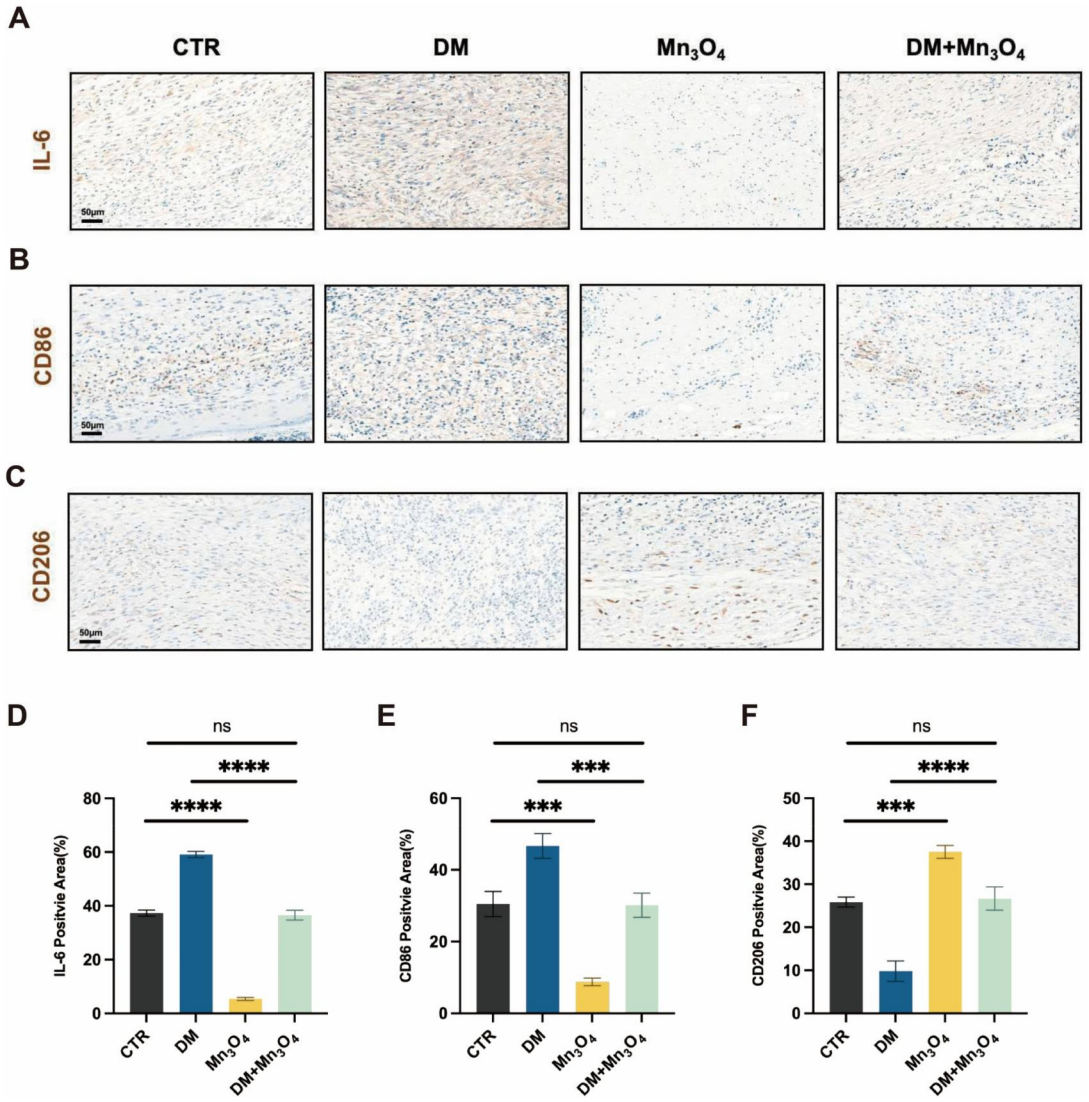
Histological evaluation and statistical analysis of diabetic wound after Mn_3_O_4_ treatment by immunohistochemical staining. Immunohistochemical staining of (**A**) IL-6, (**B**) CD86 and (**C**) CD206 in the wound bed at day 7. (**D**–**F**) Statistical results of immunohistochemical staining in each group. Data are presented as mean ± SD. Statistical significance was determined using *t*-test (*n* = 3, ns = no significance, ***P < 0.001, ****P < 0.0001).

The immunohistochemical results are shown in Figure 8A–C, where the brown-yellow color represents positive areas. It can be seen that IL-6 positivity was most pronounced in the DM group. Additionally, both the CTR group and the DM+Mn_3_O_4_ group showed some positive expression, while the Mn_3_O_4_ group showed significantly less. The statistical analysis, as shown in Figure 8D, revealed that Mn_3_O_4_ nanozyme significantly reduced IL-6 expression in wounds of both normal and diabetic mice, suggesting that the nanozyme effectively alleviated the inflammatory response. As shown in Figure 8E and F, compared with the CTR group, the DM group had upregulated CD86 and downregulated CD206, while the Mn_3_O_4_ group showed the opposite pattern with downregulated CD86 and upregulated CD206. Furthermore, in the DM+Mn_3_O_4_ group, compared with the DM group, the addition of Mn_3_O_4_ nanozyme significantly decreased the CD86-positive area and increased the CD206-positive area, indicating a reduction in M1 macrophages and a corresponding increase in M2 macrophages. These suggested that Mn_3_O_4_ nanozyme promoted the shift of macrophages from M1 to M2 polarization *in vivo*, thereby reversing the chronic inflammatory state in diabetes and promoting tissue healing. Additionally, immunofluorescence staining (Supplementary Figure S9) revealed significantly elevated CD31 expression in the Mn_3_O_4_ group and DM+Mn_3_O_4_ group compared to the H_2_O_2_ group at day 7. Conversely, the DM group exhibited negligible CD31 expression, indicating impaired neovascularization. These results demonstrated that Mn_3_O_4_ nanozyme can facilitate early-stage vascularization and promote both neovascularization and re-epithelialization in diabetic wounds.

## Conclusion

The chronic healing of diabetic wounds is associated with elevated ROS levels and a prolonged inflammatory state in the microenvironment. To address this clinical issue, this study prepared ultra-small Mn_3_O_4_ nanozyme to promote diabetic wound healing and demonstrated its structural integrity and good SOD- and CAT-like enzymatic activities. Mn_3_O_4_ nanozyme exhibits good biocompatibility across different cell types. As the functional core of Mn_3_O_4_ nanozyme, its ROS scavenging capability is fundamental to its effectiveness. *In vitro* experiments demonstrated that Mn_3_O_4_ nanozyme can effectively alleviate intracellular oxidative stress levels and enhance the anti-inflammatory M2 polarization phenotype of macrophages, potentially through the NF-κB signaling pathway. Meanwhile, it was verified *in vitro* that M2 polarization of macrophages can alleviate the impairment of fibroblast migration and endothelial cell tube formation caused by oxidative stress. Animal experiment results also suggested that Mn_3_O_4_ nanozyme can promote wound healing by facilitating M2 polarization of macrophages in diabetic mice. In summary, the Mn_3_O_4_ nanozymes prepared in this study show great potential for the treatment of chronic diabetic wounds, and their safety and efficacy provide a new option for wound management.

## Supplementary Material

rbaf089_Supplementary_Data
