# Supplementary material for: ROS scavenging Mn3O4 nanozyme regulated immune microenvironment and affects intercellular interaction to promote wound healing in diabetes
Source: Regen Biomater. 2025 Aug 23;12:rbaf089. doi: 10.1093/rb/rbaf089 (PMC12490823; doi:10.1093/rb/rbaf089)
Supplement: rbaf089_Supplementary_Data [file rbaf089_supplementary_data.docx]

**Support Materials**


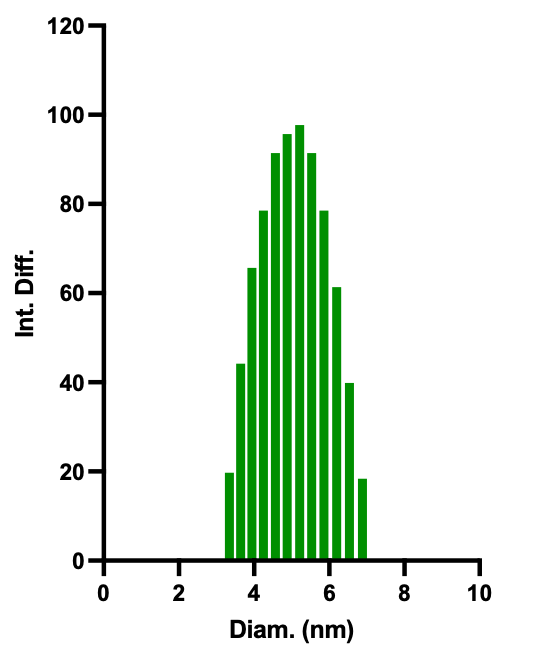


**Figure S1 The particle size analysis of Mn_3_O_4_ nanozyme.**

**
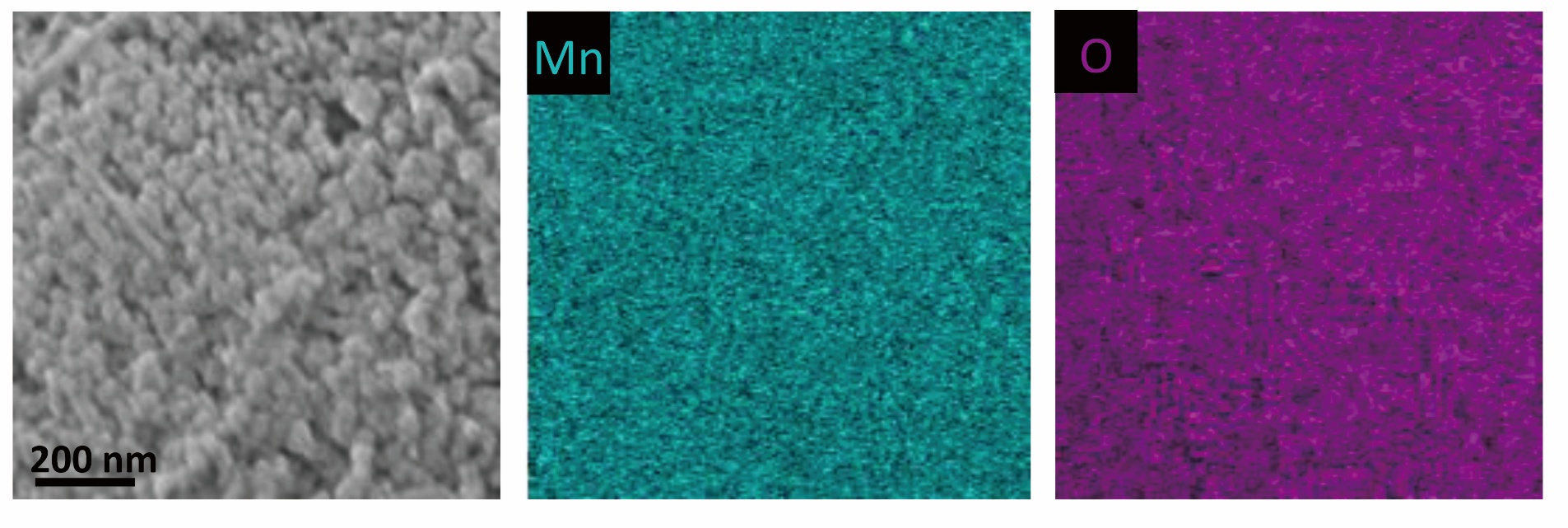
**

**Figure S2 SEM images and EDS mapping images of Mn_3_O_4_ nanozyme.**

**
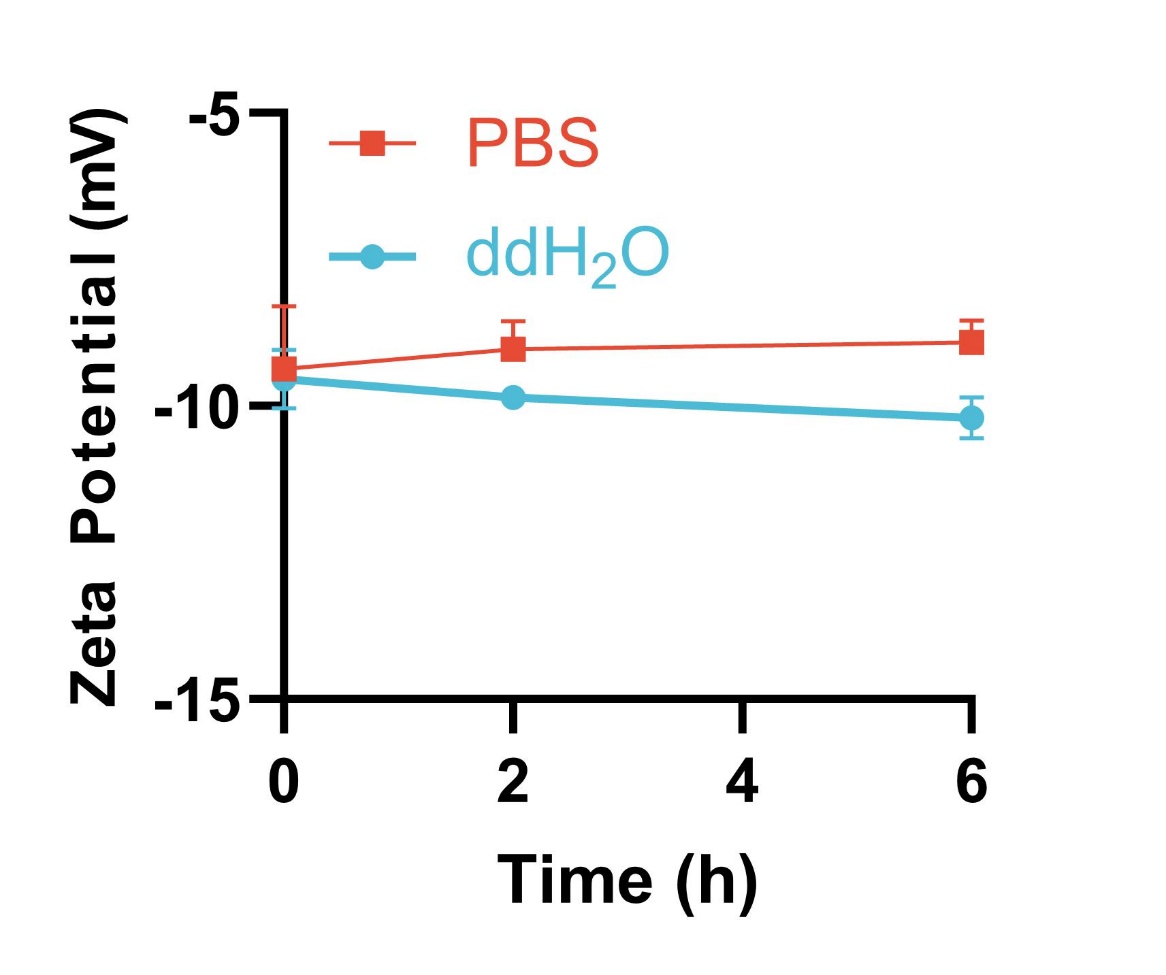
**

**Figure S3 Zeta potentials of Mn_3_O_4_ nanozyme.**

**
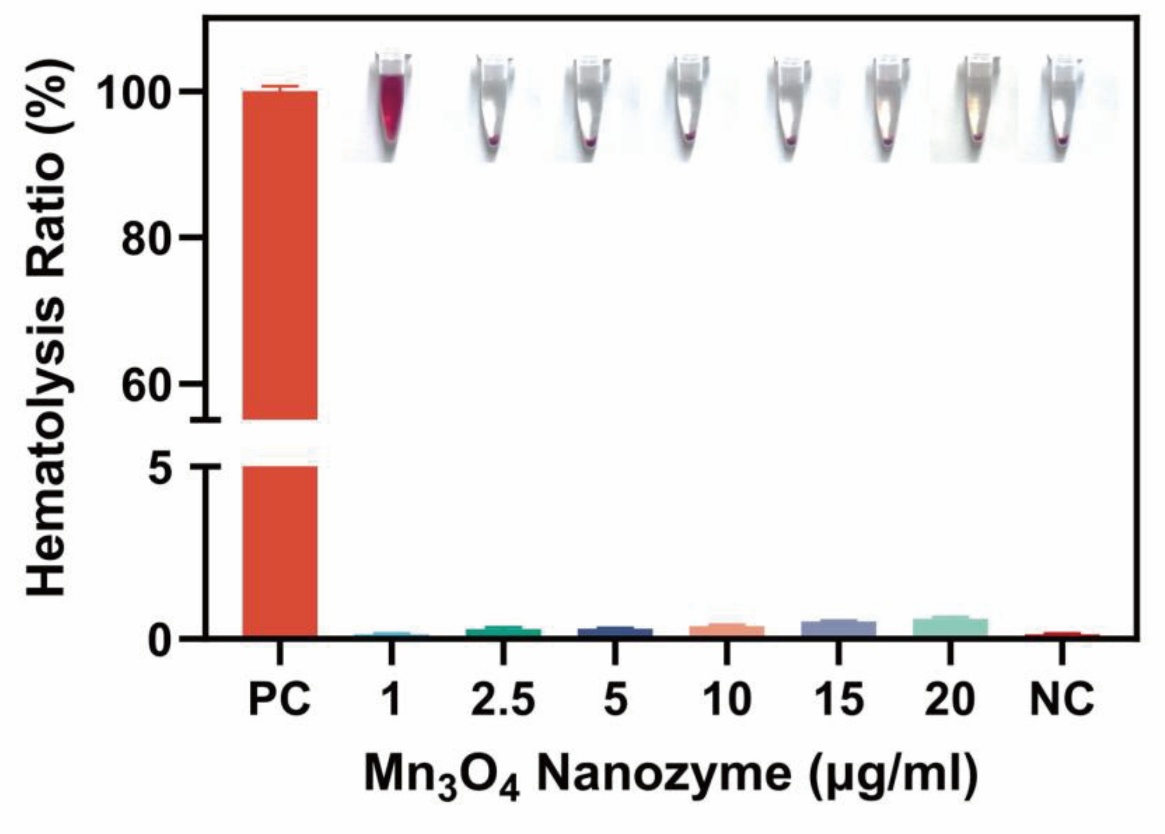
**

**Figure S4 The hemocompatibility of Mn_3_O_4_ nanozyme.**

**
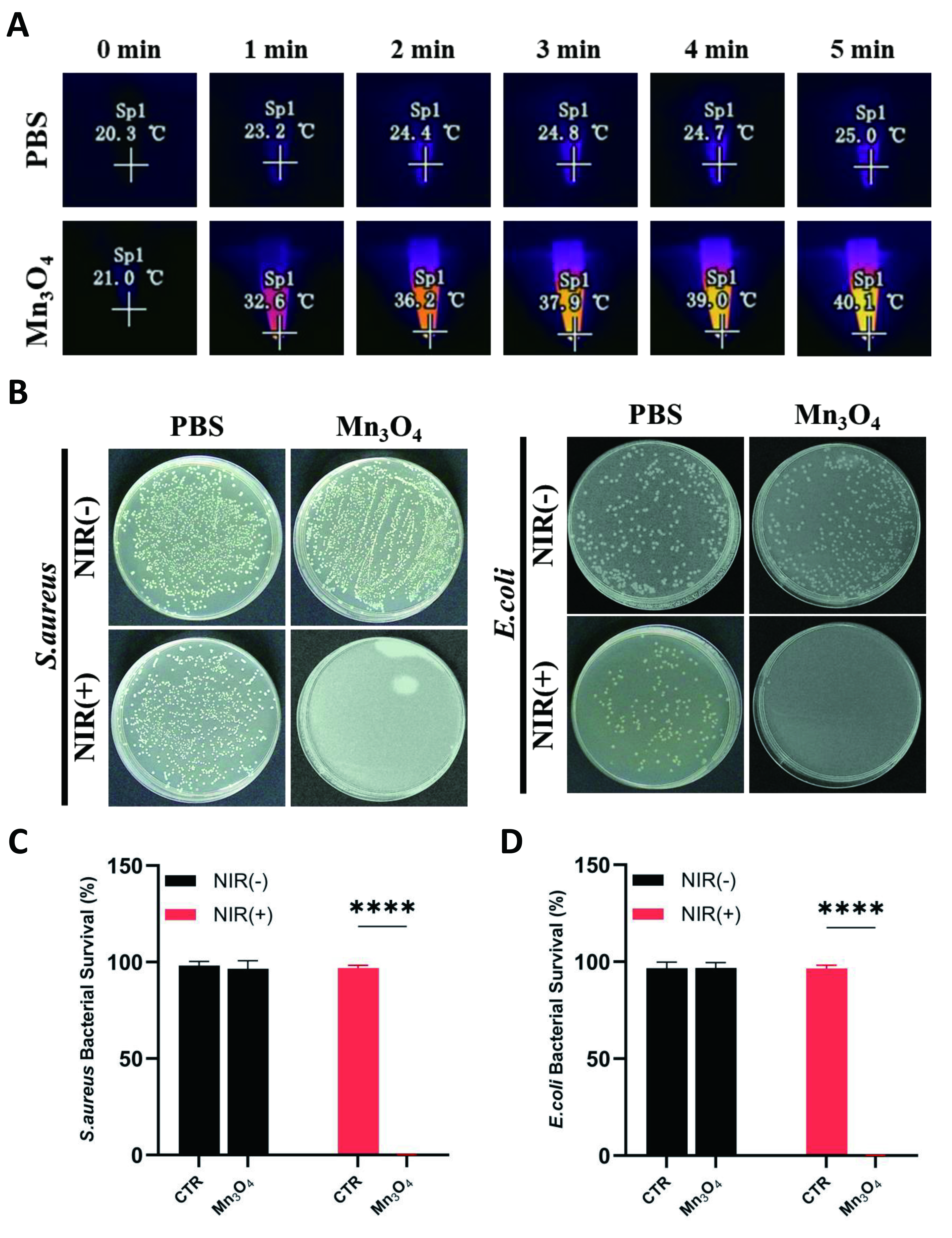
**

**Figure S5 Photothermal performance measurements and *in vitro* antibacterial capability of** **Mn_3_O_4_ nanozyme. (A) Thermal images of Mn_3_O_4_ nanozyme (10 μg/ml) under 808 nm NIR irradiation (2.0 W cm^-2^), (B) Optical images of *S. aureus* and *E. coli* bacterial colonies after treatments *via* Mn_3_O_4_ nanozyme with or without NIR irradiation, (C-D) The corresponding bacterial survival ratio results.** **Data are presented as mean ± SD. Statistical significance was determined using t-test (n=3, **** p < 0.0001).**

**
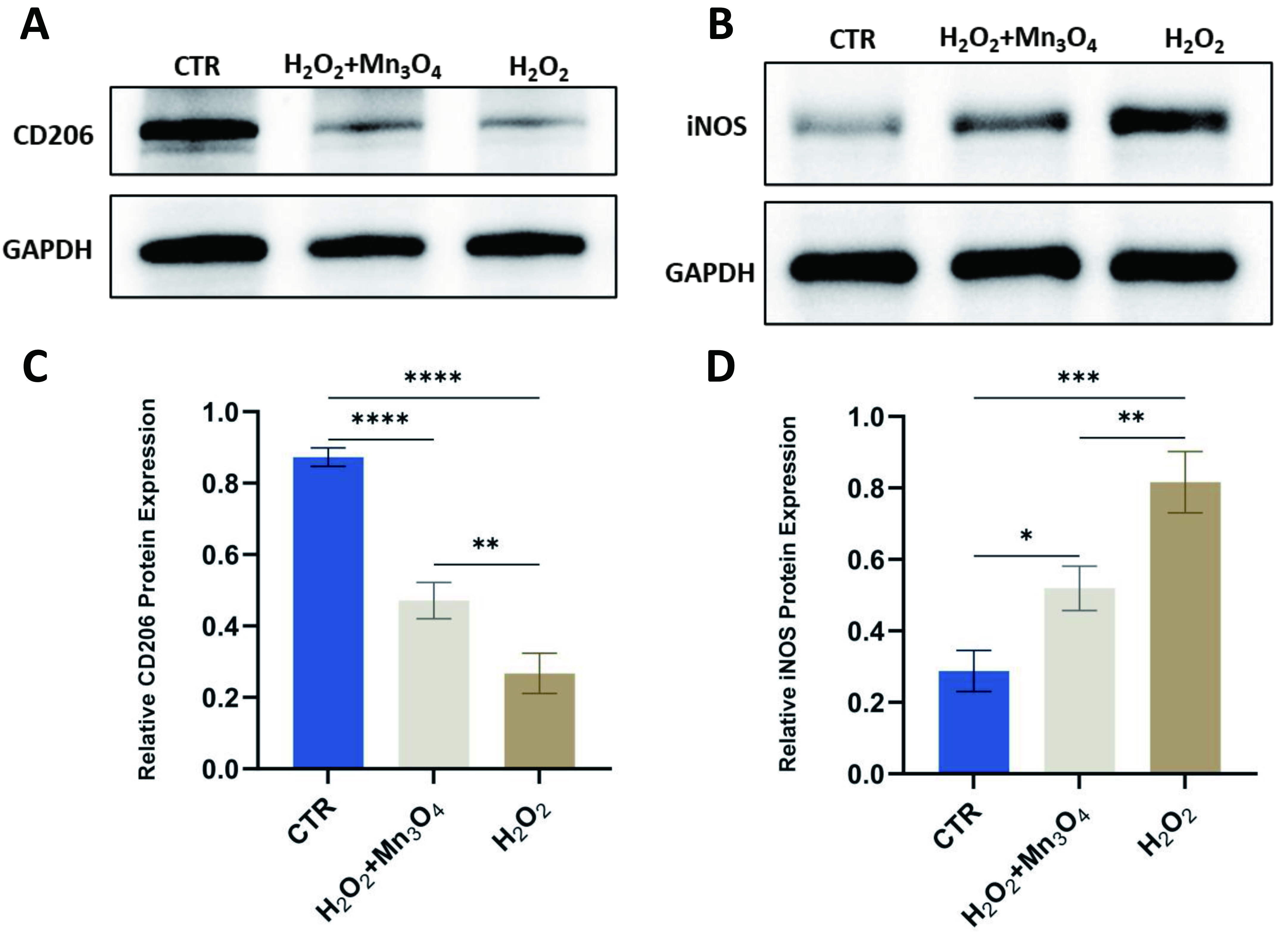
**

**Figure S6 WB results of the phenotypes of macrophages (A-B) WB analysis of CD206 and iNOS expression in RAW 264.7 cells, (C-D) The relative protein expression of CD206 and iNOS. Data are presented as mean ± SD. Statistical significance was determined using t-test (n=3, * p<0.05, ** p < 0.01, *** p<0.001, **** p < 0.0001).**

**
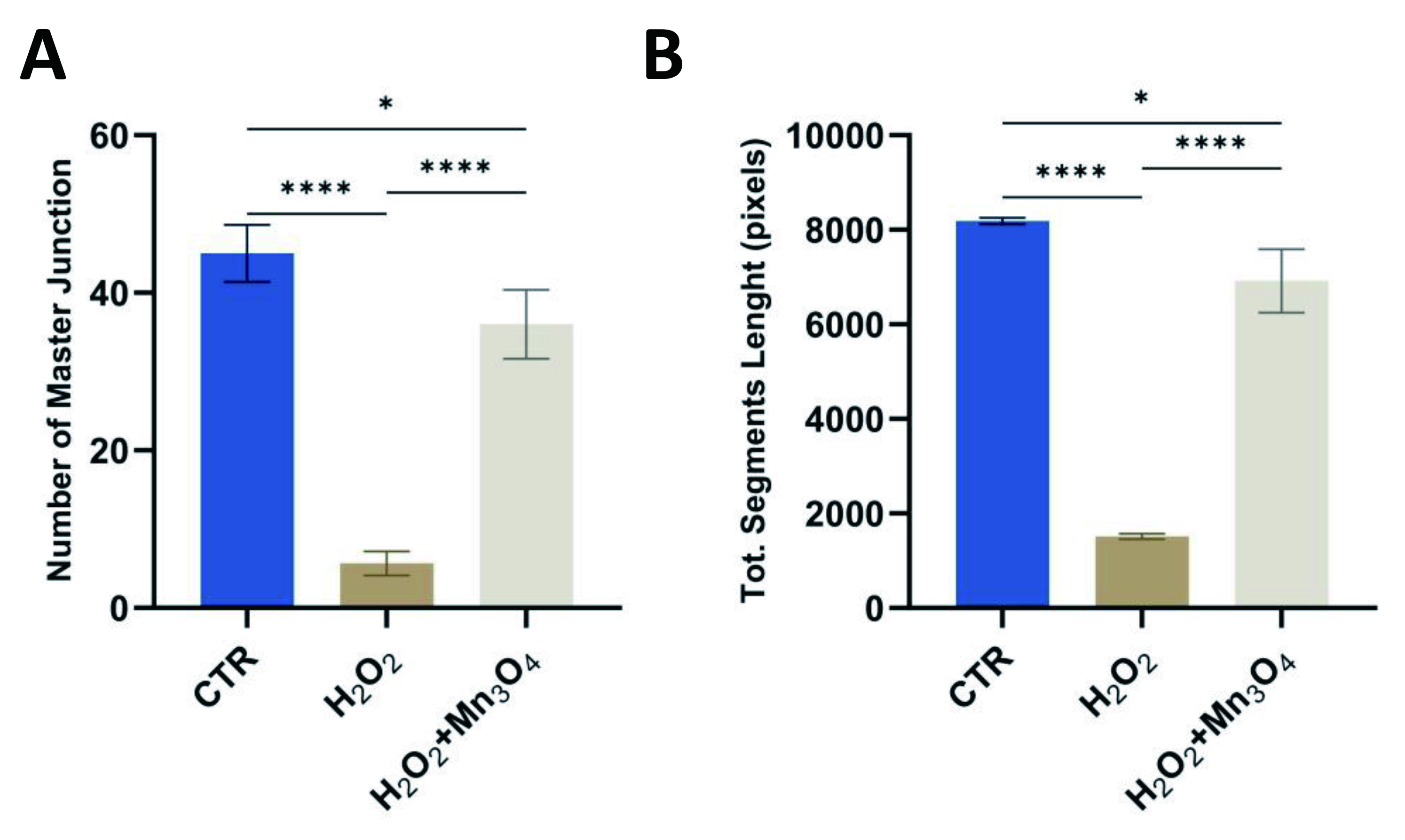
**

**Figure S7 The corresponding (A) number of master junction and (H) total segments length of tube formation assay. Data are presented as mean ± SD. Statistical significance was determined using t-test (n=3, * p<0.05, **** p < 0.0001).**

**
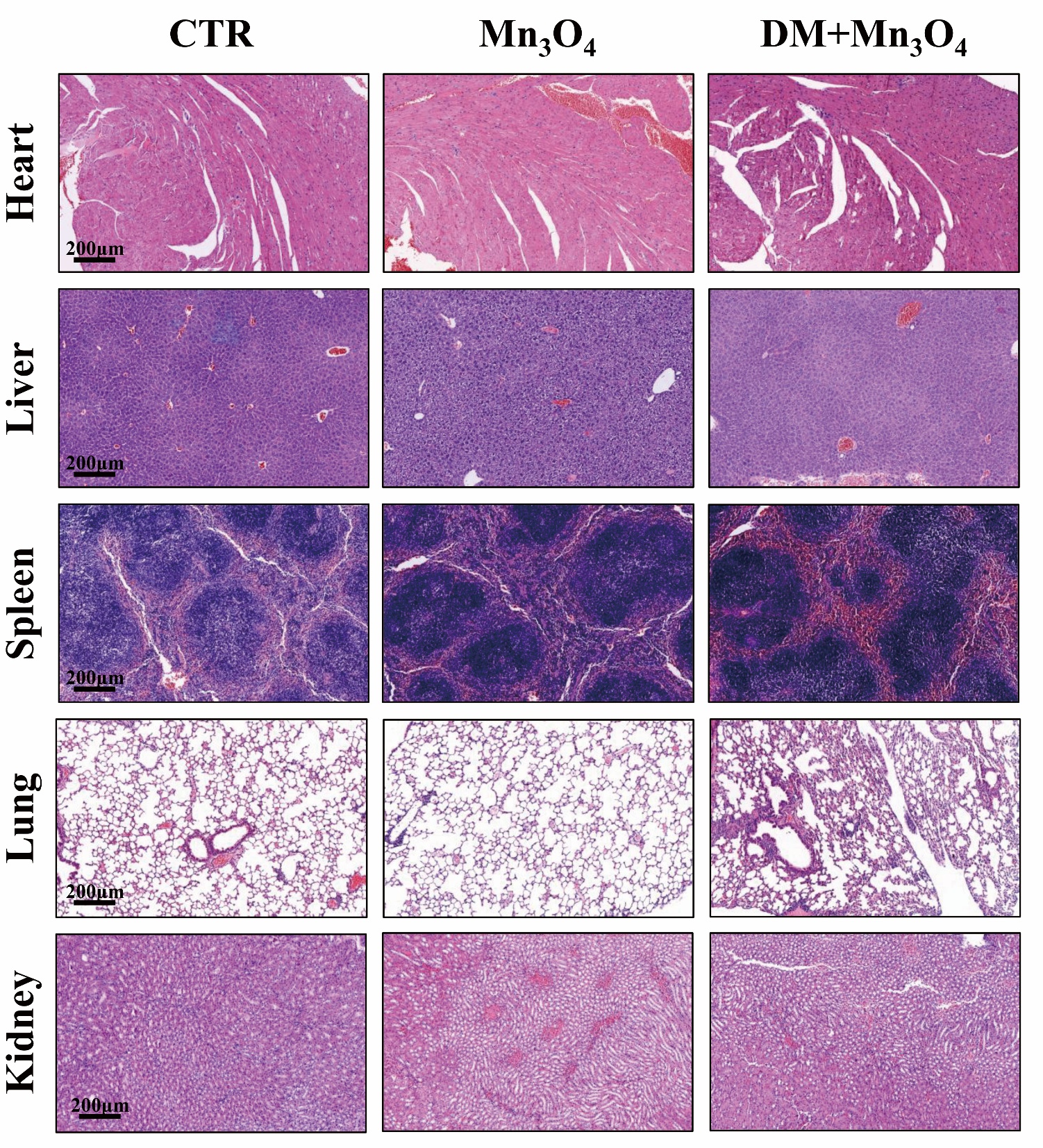
**

**Figure S8 H&E staining of major organs (heart, liver, spleen, lung and kidney) of mice on day 14 in different groups.**

**
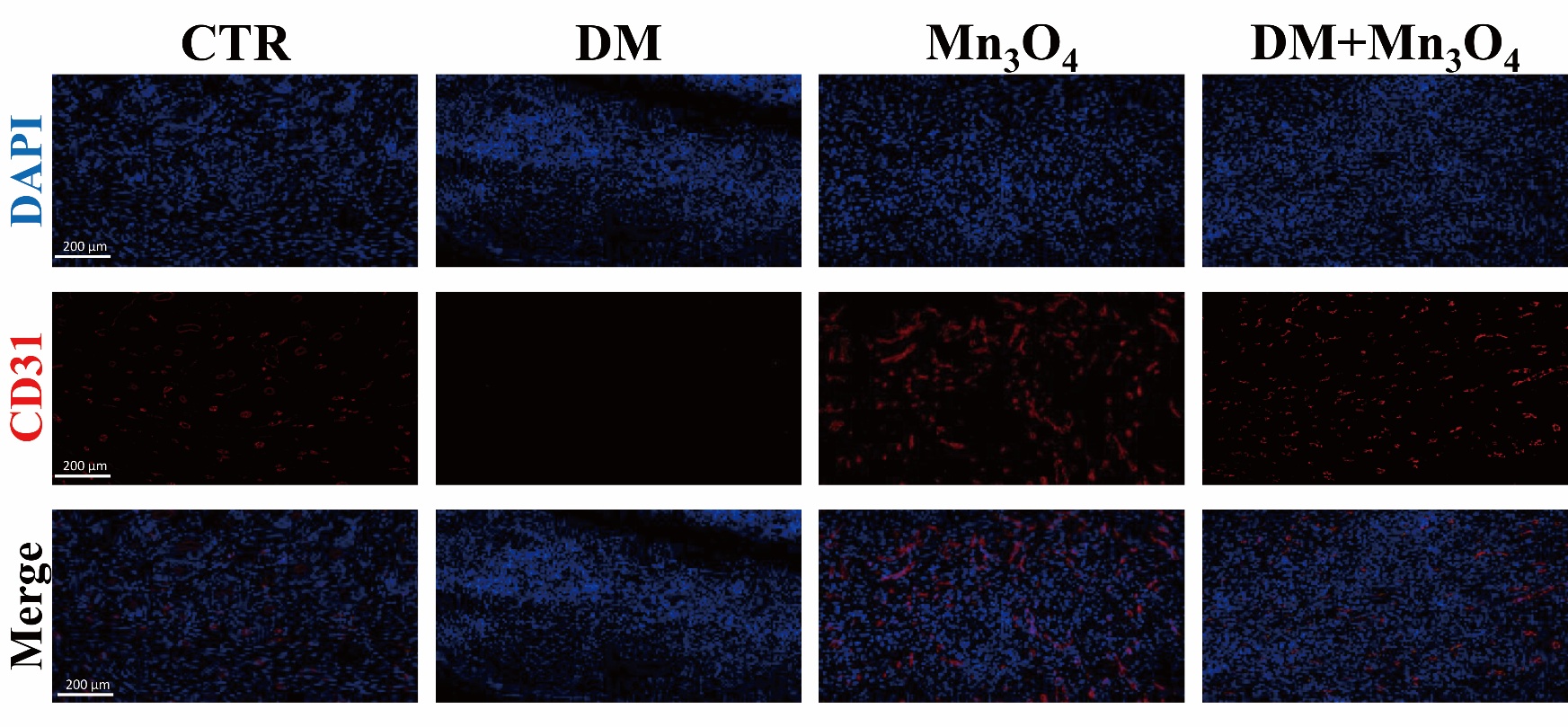
**

**Figure S9 Immunostaining of CD31 on day 7.**

**Table S1 Detecting Primer Sequence**

| **Gene** | **Sequence** |
| --- | --- |
| **TNF-α** | **F: CCTCTTCTCATTCCTGCTTG**  **R: GTCACCCCGTCCACATCTT** |
| **IL-6** | **F: TTGCCTTCTTGGGACTGAT**  **R: TTGCCATTGCACAACTCTT** |
| **iNOS** | **F: AGAGCCACAGTCCTCTTTGC**  **R:CTGGTCCATGCAGACAACCT** |
| **IL-10** | **F: TGAATTCCCTGGGTGAGAAG**  **R: TGGCCTTGTAGACACCTTGG** |
| **Arg1** | **F: CCACAGTCTGGCAGTTGGAAG**  **R: GGTTGTCAGGGGAGTGTTGATG** |
| **TGF-β** | **F: TTTCCGCTGCTACTGCAAGTC**  **R: AGGGCTGTCTGGAGTCCTCA** |
| **GAPDH** | **F: CATCACTGCCACCCAGAAGACTG**  **R: ATGCCAGTGAGCTTCCCGTTCAG** |

**Table S2 Differential expression gene statistics**

| **Gene** | **log2FoldChange** | **p value** |
| --- | --- | --- |
| **IL-6** | **11.2021** | **5.7425×10^-21^** |
| **IL-1β** | **9.6258** | **0** |
| **IL-1α** | **8.9149** | **0** |
| **MMP9** | **5.0988** | **0** |
| **TNFα** | **3.1947** | **2.5782×10^-285^** |
| **CD86** | **2.6753** | **6.4763×10^-98^** |
